# In Situ X‐Ray Tomography and Acoustic Emission Monitoring of Damage Evolution in C/C‐SiC Composites Fabricated by Liquid Silicon Infiltration

**DOI:** 10.1002/advs.202516200

**Published:** 2025-11-13

**Authors:** Yang Chen, Renato S. M. Almeida, Stefan Flauder, Guillaume Couégnat, Kamen Tushtev, Jürgen Horvath, Peter Wriggers, Kurosch Rezwan

**Affiliations:** ^1^ Centre for Integrated Materials Processes and Structures Department of Mechanical Engineering University of Bath Claverton Down Bath BA2 2AY UK; ^2^ Advanced Ceramics Universität Bremen Am Biologischen Garten 2 28359 Bremen Germany; ^3^ Chair of Ceramic Materials Engineering (CME) University of Bayreuth Prof.‐Rüdiger‐Bormann‐Str. 1 D‐95447 Bayreuth Germany; ^4^ LCTS (Univ. de Bordeaux/CNRS/CEA/SAFRAN CERAMICS) 3 allée de la Boétie Pessac F‐33600 France; ^5^ Institute of Continuum Mechanics Leibniz Universität Hannover An der Universität 1 30823 Garbsen Germany; ^6^ MAPEX – Center for Materials and Processes University of Bremen 28359 Bremen Germany

**Keywords:** acoustic emission, damage evolution, liquid silicon infiltration, residual silicon, X‐ray computed tomography

## Abstract

Carbon fiber‐reinforced silicon carbide (C/C‐SiC) ceramic matrix composites are attractive materials for high‐temperature applications due to their excellent thermal and ablation resistance. This paper investigates how the morphology of residual silicon affects damage behavior in C/C‐SiC composites fabricated via the Liquid Silicon Infiltration (LSI) process using thermoplastic precursors. Two model materials are examined: one with interconnected silicon and another with isolated silicon. In situ X‐ray computed tomography (XCT) is combined with acoustic emission (AE) monitoring during tensile testing. The multi‐modal approach enables full‐field strain mapping and real‐time tracking of damage evolution. The results reveal that the distribution and connectivity of the silicon bulks significantly influence local strain distributions and crack development, features that are not accessible through conventional macroscopic testing. This detailed study demonstrates the value of integrating XCT and AE data into a unified damage analysis framework to overcome the limitations of each in isolation. This work provides a first microstructure‐level investigation on how residual silicon and its local arrangement influence damage behavior of C/C‐SiC composites. The new insights gained here contribute to an in‐depth understanding of microstructure‐performance relationships in C/C‐SiC composites, highlighting pathways for optimizing LSI processing parameters to enhance damage tolerance.

## Graphical abstract

This study investigates how the internal structure of fiber‐reinforced ceramic composites affects their resistance to damage. By combining 3D X‐ray imaging with acoustic emission monitoring during mechanical testing, it reveals how silicon distribution influences crack formation. The findings offer new insights for designing tougher, more reliable materials for high‐temperature engineering applications.

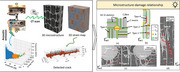

## 1 Introduction

Ceramic matrix composites (CMCs) provide unparalleled material performance for high‐temperature applications in critical industry sectors, such as aerospace and nuclear energy. A notable concept involves C/C‐SiC composites, fabricated via the Liquid Silicon Infiltration (LSI) process. These materials offer a compelling combination of high‐temperature resistance and remarkable ablation resistance, which makes them ideal for applications like spacecraft heat shields and passenger car brake disks.^[^ ^1^, ^2^, ^3^, ^4^ ^]^ The fabrication of C/C‐SiC composites involves infiltrating melted silicon into a porous C/C composite, known as a green body, which is itself produced from the pyrolysis of a carbon fiber reinforced polymer composite (CFRP). During this initial process, the thermal residual stresses within the green body are believed to be largely, though not entirely, released due to the formation of micro‐cracks within fiber tows. These micro‐cracks, along with macro‐pores (pores between tows), are subsequently filled by liquid silicon during the LSI process at temperatures up to 1600 °C. This high temperature ensures a low viscosity, high chemical reactivity, and good wetting of the green body, allowing silicon to react with the carbon matrix and form a SiC matrix. The LSI process typically yields a composite with low porosity, usually in the range of 1–4%.^[^ ^5^ ^]^ However, this in situ chemical reaction induces high internal stresses, potentially leading to the formation of weak points, especially at the interfaces between constituents. These weak points are thought to be key mechanisms contributing to the initial nonlinear stress–strain behavior of the composites, a characteristic specifically designed to improve toughness.^[^ ^6^ ^]^

The initial fabrication route for LSI C/C‐SiC composites was based on CFRP with a thermosetting matrix.^[^ ^5^ ^]^ To overcome the long curing cycles required for thermosetting polymers like phenolic resins, a thermoplastic matrix can be used.^[^ ^7^ ^]^ The use of a thermoplastic matrix leads to a liquid phase pyrolysis and the formation of distinctive Si‐agglomerations within the C/C‐SiC microstructure.^[^ ^7^ ^]^ It was found that C/C‐SiC derived from thermoplastics, instead of phenolic precursors, exhibits higher strength and less scattering of the strength values attributed to the difference between liquid and solid state pyrolysis during the LSI process.^[^ ^8^ ^]^ The re‐melting of the thermoplastic matrix during the LSI process can be used to introduce a new level of structure in the C/C state in terms of tailored macro‐pores.^[^ ^9^ ^]^ The size and shape of the macropores can be set from isolated pores to interconnected tube‐like pore channels.^[^ ^9^ ^]^ Through the LSI process, bulks of Si surrounded by a SiC skeleton are formed within these macropores. Previous work demonstrated that the type of macropores in the C/C green body and the Si bulk in the C/C‐SiC composite clearly affected the mechanical behavior under bending.^[^ ^9^ ^]^ However, it is still unclear how these Si bulks affect the local stress distributions and damage evolution within the composite.

In order to understand damage evolution and the resulting mechanical behavior of composites with such complex microstructure, in situ microscopic observations are necessary. In situ X‐ray tomography (XCT) is an effective tool to address these requirements. Our previous work employed in situ X‐ray tomography (XCT) imaging to characterize the internal strain distributions in a C/C‐SiC material manufactured from a CFRP with phenolic resin.^[^ ^6^ ^]^ Notably, no macropores were present in the C/C green body, and thus the final microstructure did not have any Si bulks between fiber tows. This full‐field characterization approach has subsequently been adopted in several recent studies involving similar yet distinct C‐SiC composite systems, e.g.^[^ ^10^, ^11^, ^12^, ^13^ ^]^ to name a few. A trend in these efforts is to conduct such in situ experiments under extreme conditions, particularly at elevated temperatures. In addition to various microstructural analyses based on image processing, 3D strain fields can be measured using the Digital Volume Correlation (DVC) technique. These 3D measurements provide quantitative information on the heterogeneous deformation of the material, which is strongly correlated to its microstructure. A limitation of this 3D observation approach is related to the image resolution. Typically, XCT images used in such experiments have resolutions of 1–10 µm voxel^−1^, which are high enough to capture major cracks that open widely, but insufficient to detect those with small openings. It is not yet clear whether the crack opening is proportionally correlated to the release of elastic energy, with the latter being an important indicator of damage accumulation in the material system.

Integrating acoustic emission (AE) monitoring to in situ XCT experiments enables simultaneous and complementary tracking of internal damage evolution. This multi‐modal “seeing‐and‐hearing” approach offers a more comprehensive understanding of material degradation mechanisms and is becoming increasingly attractive, as demonstrated by recent trends in the literature. While some studies, such as,^[^ ^14^, ^15^, ^16^, ^17^ ^]^ have paired AE with ex situ XCT scans, the simultaneous in situ approach remains largely unexplored. Maire et al.,^[^ ^18^ ^]^ applied this in situ combination to investigate the damage events within particle‐reinforced metal matrix composites. However, their work lacked a thorough and quantitative analysis of the XCT data, and the AE data analysis was limited to overall cumulative energy and AE hit counting. Chen et al.^[^ ^19^ ^]^ monitored the AE signals during a synchrotron in situ XCT experiment on braided SiC‐SiC composite tubes. While their in situ XCT data allowed for high‐quality quantitative analyses of microcracks through full‐field measurement, their AE data analysis was restricted to overall cumulative energy and hit count. Maillet et al.,^[^ ^20^ ^]^ conducted a 4‐point bending experiment on unidirectional SiC‐SiC laminates, combining in situ synchrotron XCT and AE monitoring. Although they analyzed the AE data with greater depth, including the signal locations, it was still largely confined to overall cumulative energy and hit count, and their crack quantification from XCT data was performed manually, prohibiting a full‐field measurement. More recently, Deresse et al.,^[^ ^21^ ^]^ combined these two techniques to analyze the damage modes in cementitious mortars. Their study leveraged DVC for full‐field measurements and supplemented XCT data with the location information of AE signals. The quantitative comparison between XCT and AE data was feasible in their work due to the relatively large characteristic length of the heterogeneity, hence leading to few and loud micro‐cracking events concentrated near major crack formation sites. In contrast, such quantitative analysis is more challenging for CMCs, as their tiny microscale features lead to a large number of widely dispersed micro‐cracks throughout the volume long before a macroscopic crack appears.

In the present work, we present a detailed application of in situ XCT, complemented by AE signal clustering, to analyze the damage evolution in a C/C‐SiC material fabricated from thermoplastic precursors. Two model materials were selected based on a critical processing parameter, compaction pressure, which significantly affects the connectivity of residual silicon (Si): Batch‐A, characterized by extensive interconnected bulk Si, and Batch‐B, featuring isolated and localized Si regions. This work addresses a key knowledge gap regarding how variations in microstructure and phase composition, specifically residual silicon and its local arrangement, influence damage behavior.

We demonstrate that this integrated methodology offers unique insights into the damage behavior and microstructural characteristics of the two model materials, extending beyond what can be revealed by conventional macroscopic testing and postmortem analysis. Recognizing that C/C‐SiC composites represent a particularly challenging class within CMC systems due to their intricate microstructural details (comprising more than three distinct constitutive phases, all with complex 3D geometries), this study underscores the utility by the integrated method as a valuable tool for elucidating the relationship between microstructure and damage evolution, thereby offering broader applicability to other CMC systems, including C/C, SiC/SiC and oxide‐oxide.

## 2 Experimental Section

### 2.1 C/C‐SiC Composite Fabrication and its Characteristics

The carbon fiber‐reinforced silicon carbon (C/C‐SiC) composite materials were fabricated at the Chair of Ceramic Materials Engineering in Bayreuth, following the three‐step liquid silicon infiltration process (LSI),^[^ ^22^ ^]^ as briefly described below (see Ref. [9] for more details). Two composite plates were created, named Batch‐A and Batch‐B, which differed in their residual silicon content due to variations in their manufacturing parameters.

1. First, carbon fiber (HTA 40, Teijin Carbon Europe GmbH) reinforced thermoplastic composites (CFRP) were created by compression molding of plain weave fabrics (3k fiber bundles, weave style 460‐5). In this CFRP state, Batch‐A and Batch‐B had different fiber volume fractions (fvf) of 56.1% and 47.2%, respectively.
2. During the pyrolysis at 1000 °C in a nitrogen atmosphere, Batch‐A was compressed with 1 kPa pressure, while Batch‐B was compressed with 3 kPa pressure. The use of a thermoplastic matrix (polyether ether ketone, KetaSpire KT‐880, Solvay S. A., Europe) allowed the matrix to remelt during heating to pyrolysis temperature, enabling highly compacted fiber preforms to release elastic energy.^[^ ^9^ ^]^ The compaction pressures were chosen to achieve similar fvf for both samples in their C/C and final C/C‐SiC states.^[^ ^9^ ^]^ Batch‐A, with its higher initial fvf (56.1%) and subjected to a lower compaction pressure (1 kPa), experienced a greater release of stored elastic energy from its compressed woven fabric. This resulted in an observable increase in the plate thickness and the formation of more interconnected macropores in the C/C green body.^[^ ^9^ ^]^ Consequently, Batch‐A exhibited a higher open porosity in its C/C state (26.7%) compared to Batch‐B (22.4%). This open porosity consists of open crack networks from shrinkage^[^ ^23^, ^24^ ^]^ and the additional tailored macropores within the materials.^[^ ^9^ ^]^
3. The third and final step involved infiltrating the porous C/C green body with liquid silicon at ≈1600 °C. The liquid silicon rapidly fills the existing crack networks and void areas within the composites, simultaneously reacting with the amorphous carbon to form SiC. The material then undergoes slow cooling to room temperature. This process resulted in the final C/C‐SiC materials with similar porosity of ≈4.4% and a comparable fvf of 51.6% and 51.3% for Batch‐A and Batch‐B, respectively.

The phase composition of the C/C‐SiC material was determined from mass changes after oxidation and the measurement of skeletal densities with helium pycnometry. The density of the carbon phase was measured by pycnometry of milled C/C green bodies and found to be 1.66 g cm^−3^. The total amount of carbon phase (fibers and matrix) in the C/C‐SiC composites was measured from the mass loss after oxidation of milled powder at 700 °C for 9 h. The remaining powder after oxidation was composed exclusively of SiC and Si, with known densities of 3.21 and 2.33 g cm^−3^, respectively. This allowed the mass and volume fractions of each phase to be calculated, as shown in **Table** **1** alongside other geometrical and compositional characteristics.

**Table 1:** Microstructural characteristics and mechanical properties of Batch‐A and Batch‐B samples.

Batch	Number of plies	Compaction pressure [kPa]	Fvf in C/C‐SiC	Thickness [mm]	Si [Vol‐%]	SiC [Vol‐%]	C [Vol‐%]
A	11	1	51.6%	2.70	20.7%	15.7%	63.6%
B	9	3	51.3%	2.25	14.3%	16.0%	69.7%

The higher open porosity in Batch‐A at its C/C state resulted in a greater volume fraction of Si and a lower C content in the final composite. The volume fractions of carbon fibers (fvf) and SiC phase remained comparable between both batches, which should be kept in mind when interpreting the results in Section 3.

Macroscopic tensile tests were repeated four times for each batch, with experimental details in the previous work.^[^ ^25^, ^26^ ^]^ These macroscopic stress–strain curves will be compared to the in situ measurements in Section 3.2. The results revealed ultimate tensile strengths of 151.3 ± 2.0 MPa and 136.0 ± 3.7 MPa for Batch‐A and Batch‐B, respectively. Thus, Batch‐A with the interconnected and higher Si bulk content achieved a slightly higher strength with less scattering compared to Batch‐B at the same fiber volume content.

### 2.2 In Situ Experiment Combining XCT and Acoustic Emission

**Figure 1:**
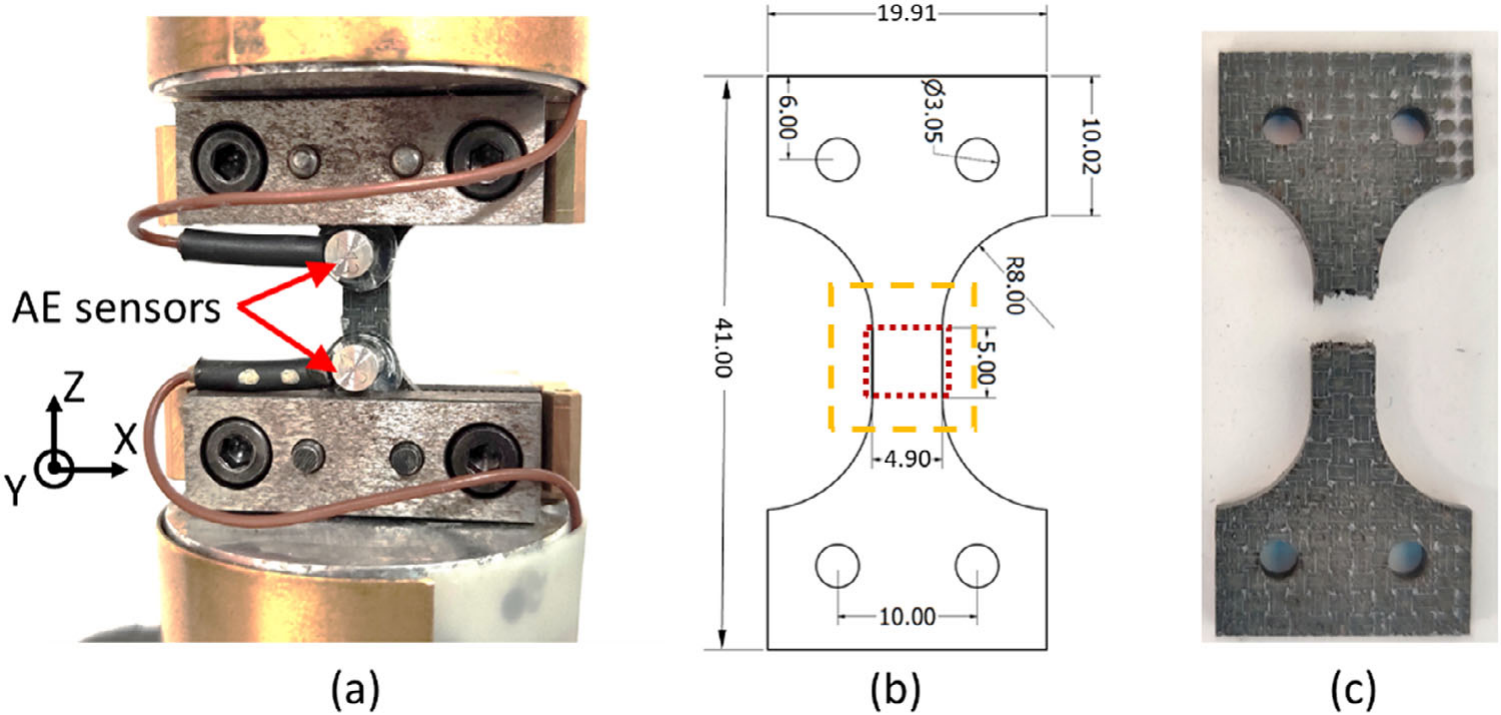
a) Sample mounted to Deben loading rig with acoustic emission (AE) sensors. The coordinate system defined in this graph will be used throughout the analyses in this paper. b) Design geometry of the dog‐bone shape samples. Dimensions are in mm. The yellow dashed and red dotted rectangles approximately indicate the XCT field of view and the sub‐region for DVC calculation, respectively. c) Sample after testing (Batch‐A).

Samples were prepared using electrical discharge machining (EDM), cut into a dog‐bone shape with pinholes to comply with the specifications of the Deben loading rig for in situ XCT experiments. This setup and sample geometry are shown in **Figure** **1**. The gauge section has a dimension of 4.9 mm × 5.0 mm to ensure that multiple woven patterns are included. Given that the size effect in CMCs is generally less pronounced compared to other materials, such as ceramics,^[^ ^26^ ^]^ such small‐sized samples could be considered representative.

The in situ experiments were performed at the University of Bremen, with an Xradia Versa 520 X‐ray microscope and a Deben CT5000‐GCT‐TEC in situ tensile rig. Acoustic emission (AE) monitoring was performed using an AMSY4‐PC system with two AE VS600‐Z2 sensors. The sample was first aligned to the loading rig using the machined pinholes and clamped with screws. The two AE sensors were directly affixed to the sample using hot glue, positioned with a center‐to‐center distance of ≈11–12 mm, as shown in Figure 1a. The use of two sensors enabled the locations of AE events to be determined along the *z*‐axis (loading direction), which were later used to filter out signals that are outside the gauge section.

Tensile tests were conducted at a loading speed of 0.1 mm min^−1^, with incremental dwells for XCT scans at 40, 100, and 125 MPa. After reaching the target loads, stress relaxation was limited to ≈50 N (3–4 MPa) for both samples. XCT scans began 5–10 min later, once the force stablized. Minor additional relaxation (≈10–20 N or 1–2 MPa) occurred during scanning, but was small enough to ensure high‐quality image reconstruction. For the X‐ray scans, an 80 kV beam energy was used without any filter applied. Each scan took 18.5 h to acquire 2401 projections, each with 25 s exposure time. The reconstructed image had a resolution of 5.3 µm voxel^−1^, with a field of view (FOV) of 11 × 11 × 11 mm^3^. The AE sensors were partially inside the FOV and caused significant artefacts in their vicinity; therefore, only the middle gauge section of the image was processed. AE monitoring was performed continuously during each loading increment with a gain of 34 dB in feature extraction mode. Hit detection was done using a threshold of 35.5 dB, a duration discrimination time of 100 µs, and a rearm time of 200 µs.

**Figure 2:**
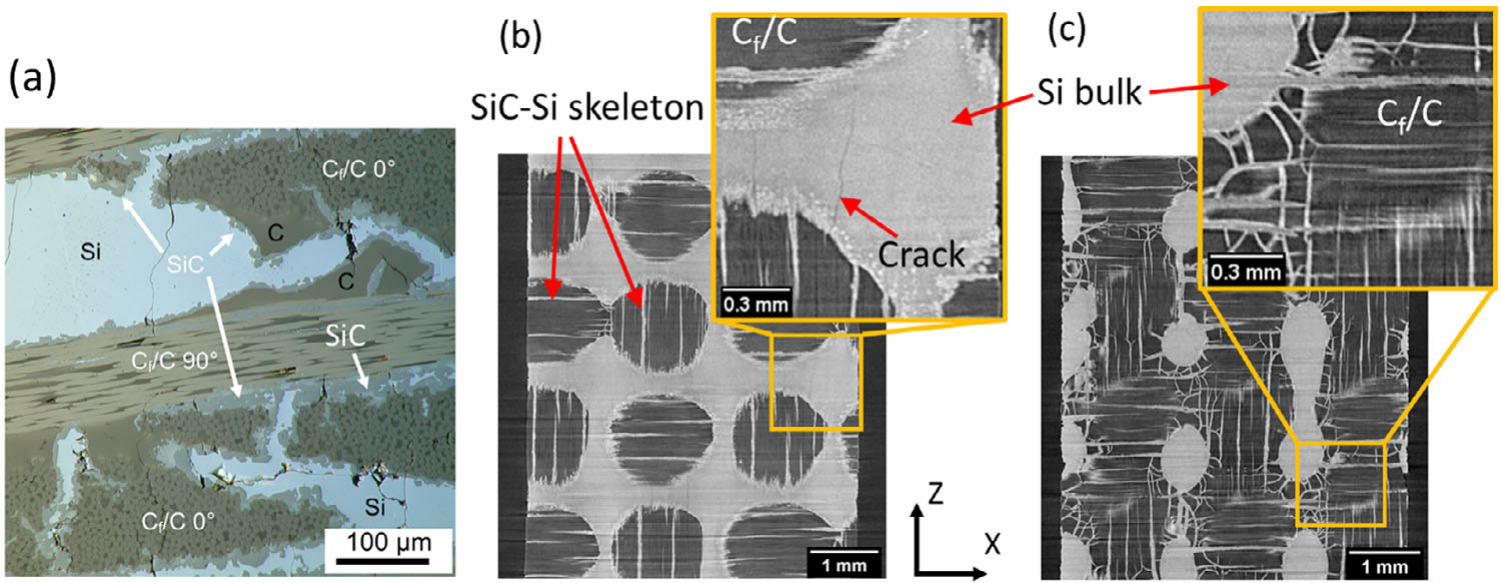
a) Optical image of the cross section of a C/C‐SiC sample (extracted from the same plate of the Batch‐B sample studied by in situ XCT), showing different phases in the composite: Si bulk (white) surrounded by SiC (pale gray), C fibers surrounded by amorphous C matrix (gray) in 0° and 90° orientation of the plain weave, and transverse microcracks (black). b,c) XCT slices in the XZ plane of Batch‐A (interconnected Si) and Batch‐B (isolated Si), respectively.

The XCT cross‐sectional slices of both samples are shown in **Figure** **2**. The SiC and Si phases exhibit similar grayscales, but are easily distinguishable from those of the carbon phase and voids. Micro‐cracks are present in both the C/C regions and the Si regions. The XCT resolution does not allow direct identification of individual C fibers. To aid interpretation, the following “mesoscale” terms are defined for the C/C‐SiC microstructure:

1. C/C block: This region contains carbon fibers and a carbon matrix. As‐manufactured micro‐cracks are present in this region, potentially formed from cooling after the siliconization stage.
2. Si bulk: This region consists of residual silicon, indicating the locations of the macropores initially present in the C/C state. Si bulks are mainly located at the places where tows cross over each other. Slightly higher grayscale appears at the periphery of Si bulk regions, indicating thin layers of SiC, which is proven by the optical image in Figure 2a.
3. SiC‐Si skeleton: This region, characterized by thin‐wall geometries, contains both SiC and residual silicon. These skeletons are primarily located inside fiber tows, suggesting that they formed within the cracks from the pyrolysis process.

The XCT cross sections in Figure 2b,c confirm the difference in residual silicon regions between the two samples. Batch‐A contains more interconnected Si bulks, whereas they are more isolated in Batch‐B. It should be noted that the presented slices in Figure 2b,c represent areas of maximum Si bulk connectivity. Additionally, the density (in number) of SiC‐Si skeletons seems higher in Batch‐B than in Batch‐A, exhibiting fibrous‐root‐like dendrites connected to the isolated Si bulks. Detailed analyses of these XCT images will be presented later in Section 3, using the techniques described in Sections 2.3–2.5.

### 2.3 Image Processing: Microstructural Analysis

The 3D image was segmented with the following steps: i) binarization with Otsu's method to identify the SiC‐Si phase, and with visual inspection to identify the threshold for the carbon phase; ii) morphological operations, including erosion, small object removal, and dilation, to separate the Si bulk from the SiC‐Si skeleton. The erosion and dilation operations both used a ball structure element of 12‐voxel (64 µm) diameter, which means that the SiC‐Si regions with a thickness larger than 64 µm would have been identified as Si bulk. It is important to note that “SiC‐Si skeleton” and “Si bulk” are not strictly quantitative definitions, as these two regions are closely connected and lack a clear distinction. To minimize subjective biases, these two regions only be analyzed with visual inspections only in Section 3.1. Nonetheless, the morphological operation described here (erosion + dilation) provided a consistent way of artificially separating the two regions in both Batch‐A and Batch‐B, enabling a comparative analysis of their microstructures.

### 2.4 Digital Volume Correlation

Digital volume correlation (DVC)^[^ ^27^ ^]^ was used to measure the 3D displacement field from the in situ experiments. This technique consists of finding the displacement field *u*(*x*) between a reference image *f*(*x*), and a deformed image*g*(*x*), such that *u*(*x*) minimizes the norm of the correlation residual ρ(*x*):

(1) $$\rho \left(x\right)=f\left(x\right)-g\left(x+u\left(x\right)\right)$$

In the context of global DVC approach,^[^ ^28^ ^]^ the displacement field is approximated using a finite‐element (FE) basis, such that:

(2) $$u\left(x\right)=\sum_{i=1}^{n_{dof}}\phi_{i}\left(x\right)a_{i}$$

where ϕ_*i*_(*x*) are the basis functions and *a_i_* the corresponding degrees of freedom. The strain field can then be obtained by deriving the displacement field using the same FE discretization.

An additional term was added to the minimization functional to control the smoothness of the solution, which is a “mechanical” regularization based on the equilibrium gap method.^[^ ^29^ ^]^ The weight of this regularization term is controlled by a user‐defined parameter $\ell_{reg}$, termed as the regularization length. The minimization of the DVC functional was performed by iteratively determining incremental corrections δ_*a*_ to the displacement field using a Newton method, until the root mean square error of the correction was lower than 10^−4^ voxels. When DVC successfully converges, the correlation residual should be at the same order of magnitude as the intrinsic noise of the images.

**Figure 3:**
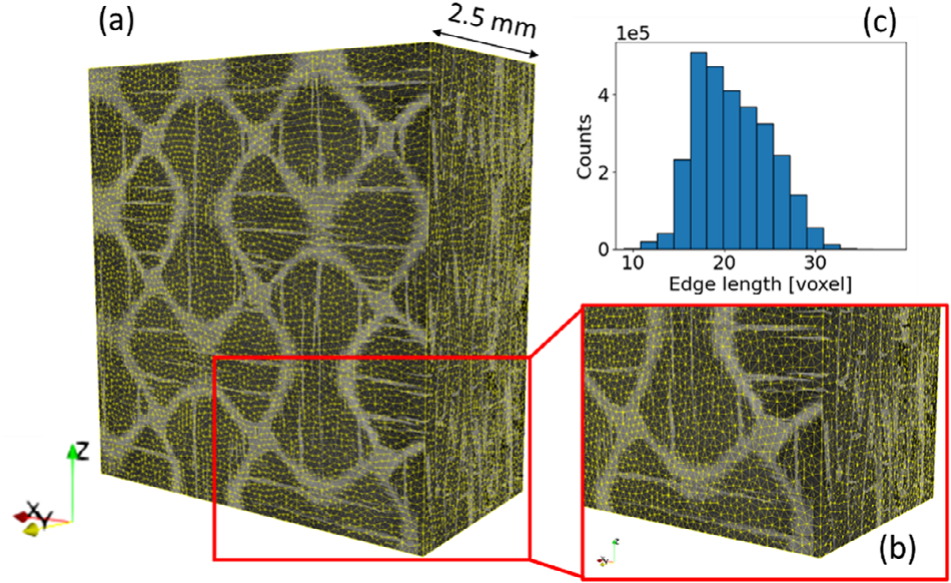
a,b) DVC mesh of tetrahedral elements for Batch‐A superimposed onto the reference grayscale image. c) Histogram of element edge lengths indicates a mode at ≈16 voxels.

The FE mesh size $\ell_{mesh}$ and the regularization length $\ell_{reg}$ influence both the spatial resolution and the uncertainty of the solution. Based on the trials (see Appendix A), an average mesh size of 16 voxels (85 µm) was chosen, leading to converged displacement fields, and a regularization length of 32 voxels (170 µm). A typical DVC mesh with tetrahedral elements is illustrated in **Figure** **3** with the histogram of the element edge lengths (indicator of element size). The edge lengths exhibited a relatively narrow distribution, with the smallest as 10 voxels and the largest as 30 voxels. To facilitate the convergence, the DVC calculation was applied in a pyramidal fashion, starting with a large regularization length (e.g., 256 voxels), and then incrementally reduced to the target value of 32 voxels. All the computations were performed using the open‐source library *kintsugi* ^[^ ^30^ ^]^ developed at LCTS Bordeaux. The resulting displacement uncertainty was estimated to be σ_*u*_ = 0.02 voxels, corresponding to a strain uncertainty of $\sigma_{\varepsilon }<10^{-3}$.

### 2.5 Crack Detection and Quantification

Correlation residuals could be further analyzed to detect crack formation within the material. This provides similar results to the image subtraction technique, as previously used in Ref ^[^ ^31^, ^32^ ^]^. The main difference here lies in the use of a global DVC approach, which provides residuals as a direct output of the method.

**Figure 4:**
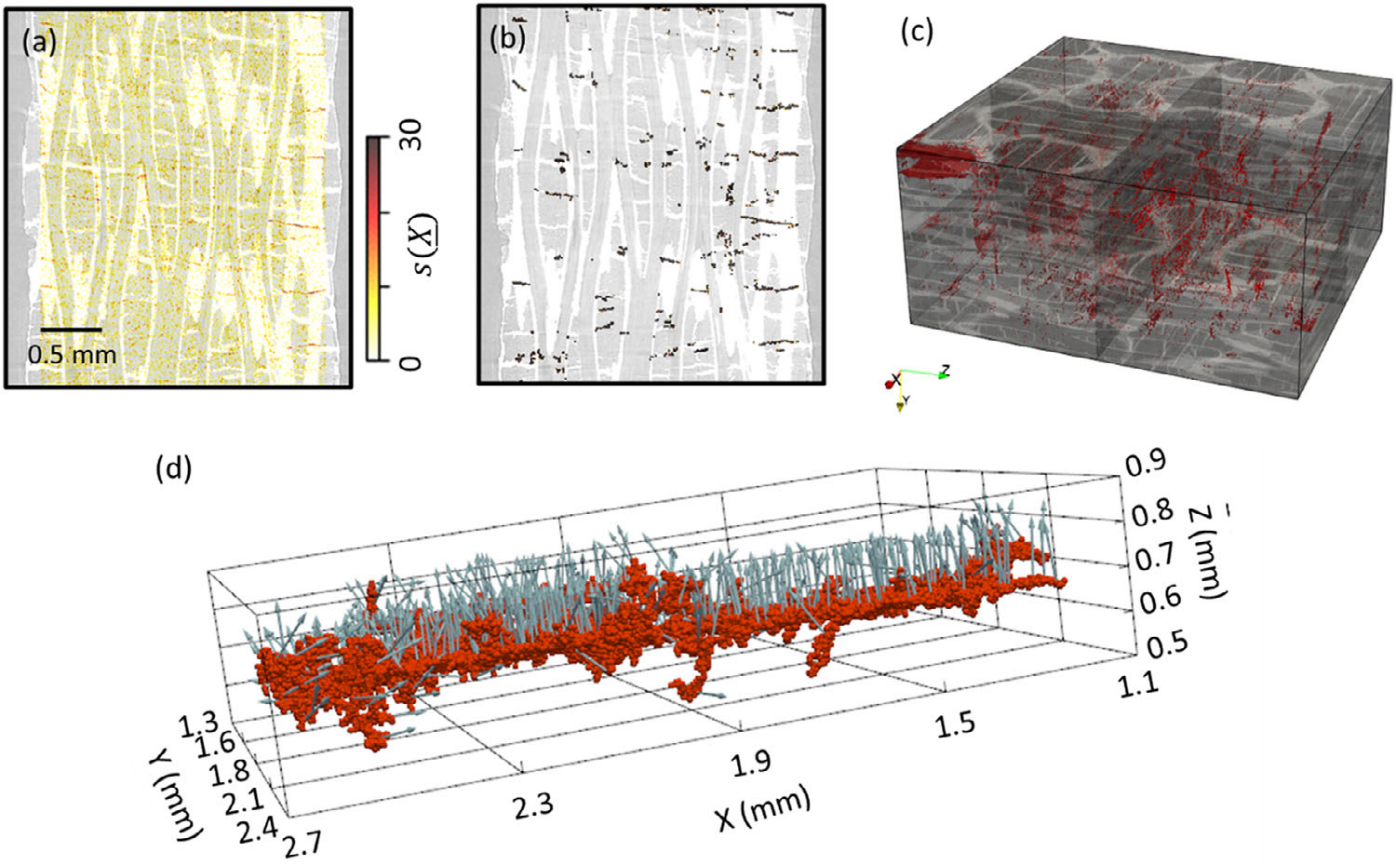
Crack detection and quantification based on in situ XCT images: a) Cross section of residual image superimposed onto the grayscale reference image. b) Detected cracks superimposed onto the grayscale reference image. c) Volume rendering of the detected cracks (red voxels), superimposed onto the grayscale reference image. d) Local orientation vectors (gray arrows) of a typical crack (red points), determined by the eigenvector of the inertia tensor (see Ref. [32] for more details).

In theory, the residuals should be comparable to the intrinsic noises from the reference and deformed images, but they could also be corrupted by image artifacts. Consequently, post‐processing was consistently required to separate the cracks from the residual noise. This procedure, along with a crack quantification method originally proposed in Ref. [32] is briefly illustrated in **Figure** **4**. First, binarization of the residuals image (Figure 4a) gives noisy detection of cracks, which are then cleaned by removing small objects (example result shown in Figure 4b). The parameters involved in this procedure were selected based on visual inspection (example shown in Figure 4c). It should be noted that cracks with volume sizes smaller than 100 voxels^3^ (530 µm^3^) could not be separated from the noise in the residual images, hence were eliminated in the final result. The inertia tensor was calculated for every crack voxel, allowing the local normal vector to be determined (Figure 4d).

### 2.6 AE Signal Clustering

**Figure 5:**
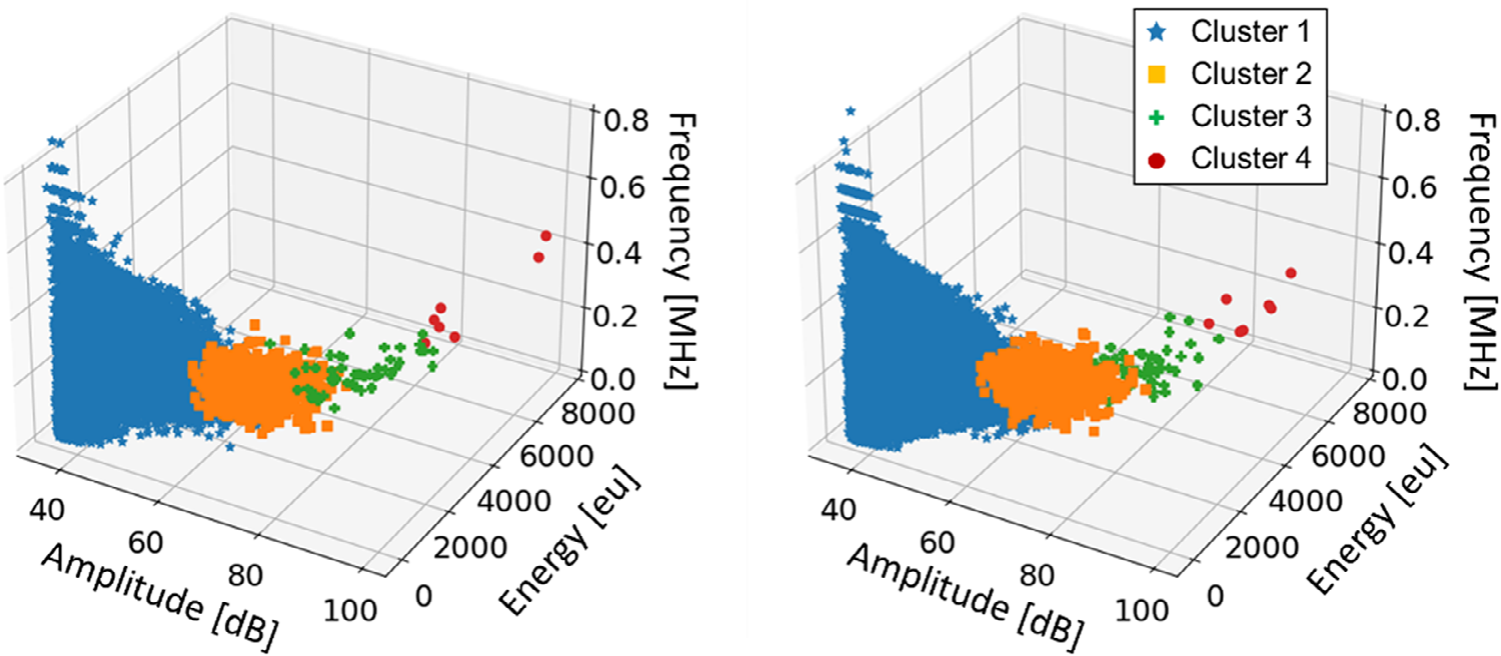
K‐means clustering of all AE signals measured during the entire in situ XCT tensile of Batch‐A (left) and Batch‐B (right).

Damage initiation and development were analyzed by acoustic emission monitoring, which was conducted continuously during the mechanical loading. Clustering of the measured AE signals was performed by the K‐Means algorithm implemented in Python using the library *sklearn*. The features used in the clustering include amplitude (dB), energy (eu = 10^−18^ J), and average frequency (MHz). Our previous work^[^ ^33^ ^]^ demonstrated that these features have a high correlation for the classification of damage mechanisms in CMCs. Before clustering, principal component analysis (PCA) was performed to reduce the dimensionality of the dataset and facilitate analysis.^[^ ^34^ ^]^ The optimal number of clusters was 4, which was determined by the elbow method–a simple and convenient visualization tool to evaluate the sum of squares within clusters using different numbers of clusters.^[^ ^35^, ^36^ ^]^ Silhouette score and Davies–Bouldin score of the clusters were measured to be 0.943 and 0.493, respectively, indicating a good clustering quality. **Figure** **5** shows the clustering of all AE signals in the feature space for both samples, with the centroids and standard deviations of each cluster given in **Table** **2**.

**Table 2:** Characteristic average (and standard deviations) of the classified clusters.

	Amplitude [dB]	Frequency [MHz]	Energy [eu]
Cluster 1	48.8 (±8.1)	0.319 (±0.115)	11 (±16)
Cluster 2	74.6 (±4.9)	0.347 (±0.057)	208 (±112)
Cluster 3	88.8 (±4.5)	0.381 (±0.067)	1125 (±437)
Cluster 4	98.3 (±2.2)	0.414 (±0.049)	5106 (±475)

Cluster 1 consists of signals with markedly lower amplitude, energy, and frequency. Hence, this cluster is associated with the cracking of the amorphous carbon matrix and the Si bulk. Cluster 2 falls within the middle range of frequency, linking it to damage at the interface between SiC and carbon matrix, particularly in regions adjacent to SiC‐Si skeletons. Clusters 3 and 4 are within the frequency range of ≈0.4 MHz, typically associated with the failure of stiff constituents, specifically the SiC phase and carbon fiber. It must be recognized that defining the physical meaning of each cluster remains a significant scientific challenge, and the above discussion is hypothetical,^[^ ^33^, ^36^ ^]^ especially as AE signals might be created by cracks that propagate through more than one phase. However, we argue that this allocation is reasonable in a statistical sense. Further experimental evidence integrating postmortem SEM analysis or machine learning models trained on labeled datasets (e.g., in Ref. [33]) could help refine these associations, yet it is beyond the scope of the present study.

## 3 Results and Discussion

### 3.1 Microstructural Analysis

**Figure 6:**
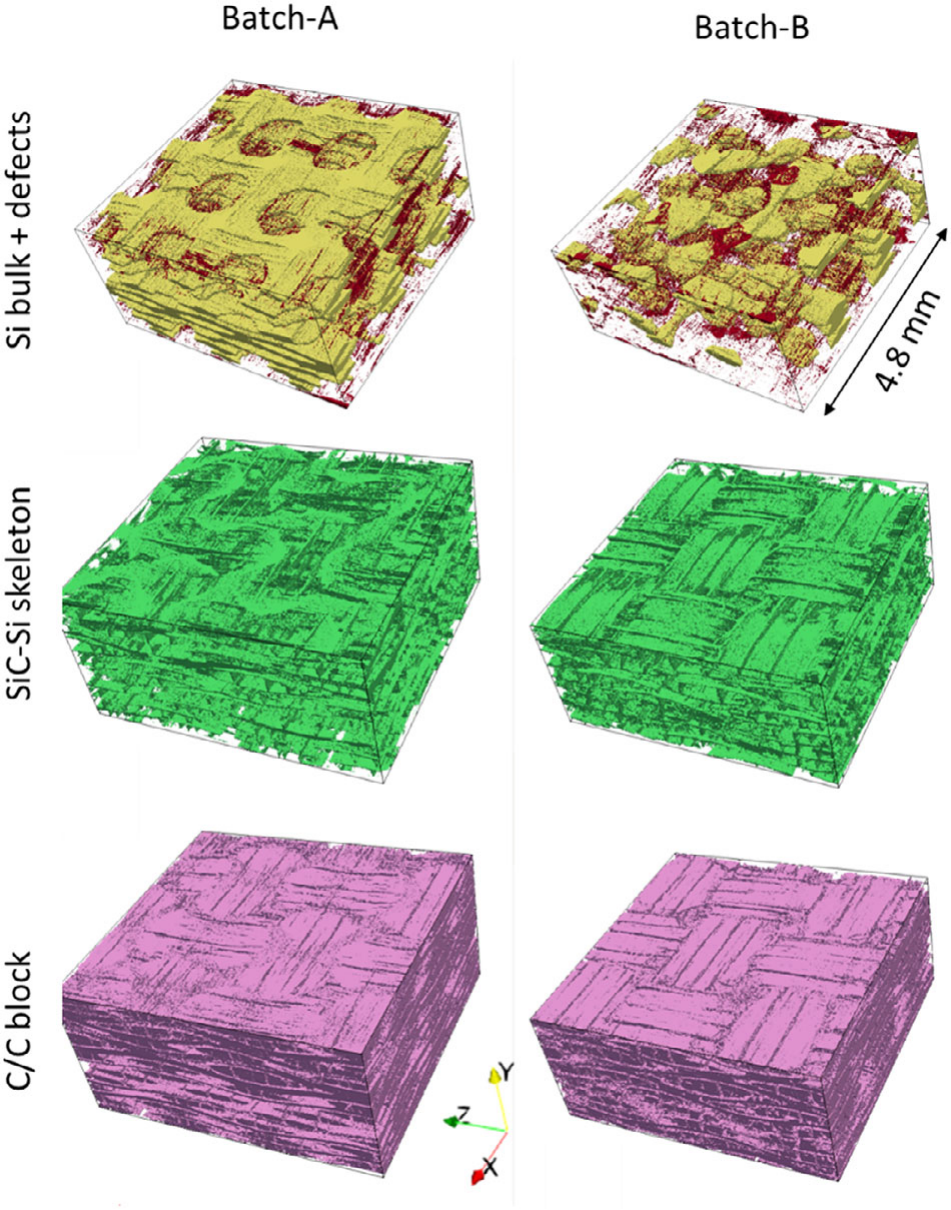
Volume renderings of the image segmentation results for the two tested samples. Different colors show the different phases, including Si bulk (yellow), SiC‐Si skeleton (green), C/C block (pink), and internal defects (red).

The as‐manufactured microstructures of Batch‐A and Batch‐B were segmented from the XCT images using the method described in Section 2.3. The results are shown in **Figure** **6**. The segmented internal defects (red voxels) are noisy, but a qualitative observation is that major defects (or initial cracks and voids) are mostly located in the cross‐over regions of yarns for both samples. These cross‐over regions might have trapped air bubbles either during the curing of the thermoplastic composites or during the silicon infiltration process. It would be interesting to conduct a porosity analysis of the initial thermoplastic composites before pyrolysis. Comparing the two samples, the overall volume fraction of Si bulk and SiC‐Si skeleton is higher in Batch‐A (38.2%) than in Batch‐B (30.2%), which is in very good agreement with the measurements from the phase composition analysis reported in Section 2.1. Figure 6 also shows the interconnected Si bulk in Batch‐A and the isolated Si bulk in Batch‐B, as a direct consequence of the fabrication process.

**Figure 7:**
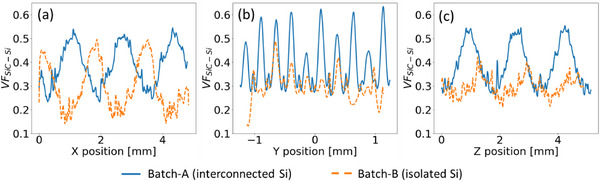
Profiles of the volume fractions of SiC‐Si phase along a) *x*‐axis, transverse direction, b) *y*‐axis, through‐thickness direction, and c) *z*‐axis, axial direction. The profiles along the *y*‐axis were re‐centered at the middle plane of the sample to facilitate the comparison between Batch‐A and Batch‐B.

The profiles of the volume fractions of the SiC‐Si phase are shown in **Figure** **7**. Both samples exhibit similar periodic patterns, with three peaks and three valleys along the transverse (*x*‐axis, Figure 7a) and the axial (*z*‐axis, Figure 7c) directions. This pattern, with a periodicity of ≈1.5–2 mm, corresponds to the plain weave structure used in the initial thermoplastic composites. It represents the width of the 3k carbon fiber bundles used in the fabric. The through‐thickness profile (*y*‐axis, Figure 7b) for Batch‐A clearly exhibit 10 peaks, while such a periodicity is less obvious for Batch‐B. In the Batch‐A profile, the number and spacing of peaks align well with the 11 ply interfaces in the sample, matching the 0.25 mm average ply thickness estimated from Table 1. The less obvious periodicity in the Batch‐B profile can be related to the higher compaction force during fabrication, creating more severe inter‐layer nesting at the interfaces. This can also explain the stronger local fluctuations in the *z*‐axis profile (Figure 7c) in Batch‐B.

### 3.2 Strain Analysis by Digital Volume Correlation

#### 3.2.1 Macroscopic Strains

**Figure 8:**
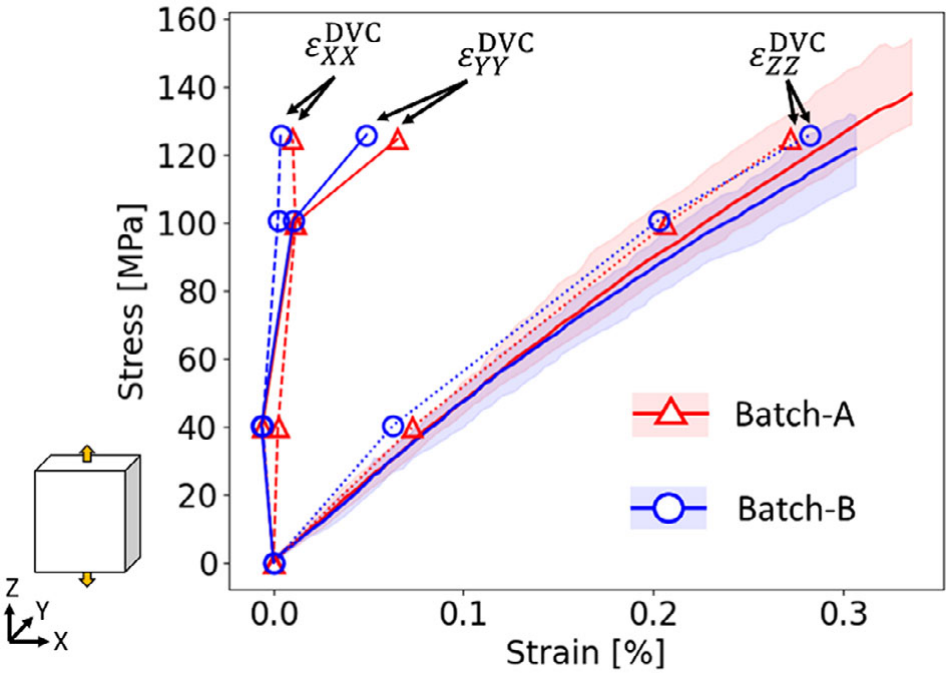
Comparison of DVC measurements (curves with markers) with stress‐strain curves measured from the macroscopic continuous tests (continuous curves). The DVC measured strain values were calculated by volume averaging each strain component over the ROI. Each continuous curve was calculated as the average of five repetition tests, with the shaded area representing their variation range. It should be noted that the continuous curves were cut slightly before the peak load to avoid a plotting issue in the shaded area.

**Figure** **8** compares the macroscopic strains measured from the in situ experiments to the stress–strain curves obtained from conventional continuous testing. The DVC measured strains (ε_*zz*_) are generally lower values than the average line of macroscopic tests, but remain within the variability range for both batches. The DVC measurements indicate the two samples exhibit similar behavior in all three strain components: the transverse strain (ε_*XX*_) remains close to zero, and the through‐thickness strain ε_*YY*_ becomes positive when the stress is high (120 MPa). This positive through‐thickness strain might be related to significant damage growth in the plane of the composite (the XZ plane), indicating delamination.

#### 3.2.2 Statistical Distribution of Strain Components

**Figure 9:**
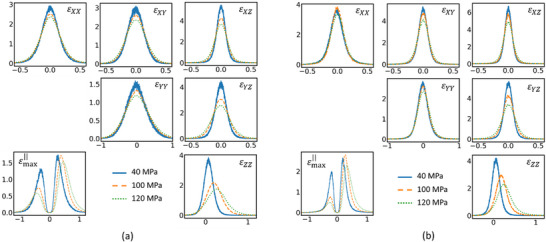
Probability density distributions of strain components ε_*XX*_, ε_*YY*_, ε_*ZZ*_, ε_*XY*_, ε_*XZ*_, ε_*YZ*_, as well as the dominant strain $\varepsilon_{\max}^{\left|\right|}$ of a) Batch‐A and b) Batch‐B at different loading steps. The strain values are expressed in percentage.

The probability density histograms of the six strain components at different loading steps are shown in **Figure** **9**. The overall trends are similar between Batch‐A and Batch‐B. The shear strains (ε_*XY*_, ε_*XZ*_, ε_*YZ*_), as well as the transverse and through‐thickness strains (ε_*XX*_, ε_*YY*_), exhibit symmetrical histograms centerd at zero, with higher variance (wider histogram profile) at higher loads. For the axial strain (ε_*ZZ*_), the histogram modes move to larger values with increasing positive skewness as the load increases. We define a quantity termed “dominant strain” $\varepsilon_{max}^{\left|\right|}$ as the principal strain that has the greatest absolute value:

(3) $$\varepsilon_{max}^{\left|\right|}=\mathrm{sign}\left(\varepsilon_{i}\right)\max_{i}\left|\varepsilon_{i}\right|$$

This quantity indicates the primary deformation mode, being either tension if its value is positive or compression if negative. As shown in Figure 9, their profiles exhibit one distribution mode in the positive range and one in the negative range, suggesting that both tensile and compressive deformation modes are found in the composites, although the macroscopic load is pure tension. The comparable size of the two distribution modes indicates that a significant number of regions undergo compression as their main deformation mode, which is not a common characteristic for a material under uniaxial tension. This should be a combined consequence of the 3D microstructures and residual thermal stresses in the C/C‐SiC composites. As the load increases, both tensile and compressive modes move monotonously toward greater absolute values, with increasing positive skewness.

#### 3.2.3 Remark

It is worth noting that the evolution of strain distributions over the increasing load observed in this material system shows distinct features compared to that of the C/C‐SiC composites studied in our previous work, which were manufactured at the DLR using CFRPs with phenolic resin. This difference is manifested by the histograms of ε_*max*_ measured at different load increments (see Figure S1, Supporting Information). In the DLR material, even very low tensile loads could create large strain values, suggesting the existence of initial weak points in the material, particularly at the interfaces between constituents. And it was observed that these initial weak points were depleted at the early loading stage. This weak point depletion is, however, not observed in the materials studied in this work–the strain histograms exhibit a mode gradually moving toward high strain magnitudes. This difference is a consequence of the significant microstructural difference: a significant amount of residual Si bulks in the present materials, which are not present in the DLR materials.

#### 3.2.4 Spatial Distribution of Principal Strains and Orientations

**Figure 10:**
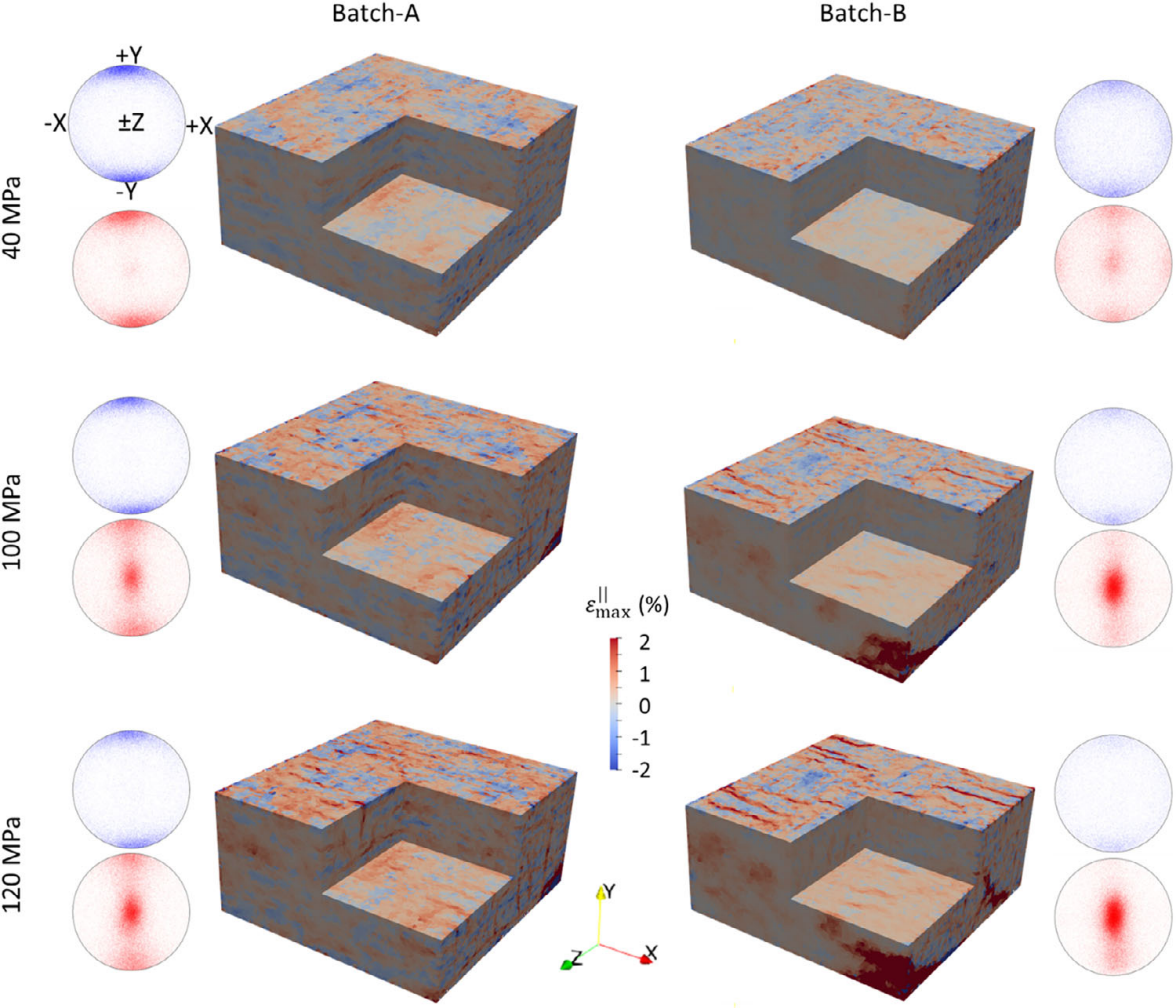
Volume rendering of the dominant strains $\varepsilon_{\max}^{\left|\right|}$ of Batch‐A and Batch‐B at different loading steps. The two polar graphs associated with each rendering represent the density maps of the orientation vectors of $\varepsilon_{\max}^{\left|\right|}$, with the blue dots representing the vectors having high‐magnitude negative strain values (within the 20th percentile of the histogram mode) and the red dots representing the high‐magnitude positive strain values (beyond the 80th percentile mode). In the polar coordinate system, the elevation describes the angle to the *z*‐axis (axial or loading direction), with 0° at the center and 90° at the perimeter. The azimuth describes the angle to the *x*‐axis, with 0°/180° (± X) in horizontal and 90°/270° (± Z) in vertical. More details on the coordinates of the polar graph can be found in Ref. [6].

The 3D volume renderings of the dominant strains are shown in **Figure** **10** for the two samples at various loading steps. Regions with high‐magnitude positive strains appear as narrow straight lines, indicating cracks perpendicular to the loading direction. These lines appear shorter and more densely spaced in Batch‐A, while in Batch‐B, they are fewer but larger. In contrast to this localized feature (narrow lines) of the positive principal strains, the negative strains have more diffuse spatial distributions in the composites. The large red regions around the corners of both samples highlight the major cracks leading to ultimate failure.

The orientation vectors associated with high‐magnitude positive and negative dominant strains are shown in the two polar graphs associated with each volume rendering. A clear distinction is observed between the two samples. In Batch‐A, both high‐magnitude negative strains (compressive mode) and high‐magnitude positive strains (tensile mode) are primarily oriented in the through‐thickness direction at 40 MPa. As the load increases, the high‐magnitude positive strains tend to re‐align with the loading direction (*z*‐axis), whereas the high‐magnitude negative strains remain in the through‐thickness direction (*y*‐axis). Contrasting with Batch‐A, the high‐magnitude strains in Batch‐B at 40 MPa did not show an obviously preferential orientation. The high‐magnitude positive strains become aligned with the loading direction as the load increases, but their negative counterpart remain relatively more random in terms of orientation.

The preferential orientation in the through‐thickness direction of the high‐magnitude strains in Batch‐A at 40 MPa suggests preliminary debonding or local delamination, likely due to the fracture of initial weak points in the material. This mechanism aligns with our previous work.^[^ ^6^ ^]^ These as‐manufactured weak points can also explain the compressive mode in Batch‐A, characterized by orientations in the through‐thickness direction. Due to the fracture of local weak points, the constituents initially deformed under tension tend to recover their stress‐free state, resulting in a compressive mode measured by the DVC. However, the above phenomenon was not observed in Batch‐B, possibly because a higher compaction pressure was applied, leading to stronger joining between the plies and/or the constituents (C/C block and Si bulk).

#### 3.2.5 Correlation Between Strain Localization, Cracks, and C/C‐SiC Microstructure

**Figure 11:**
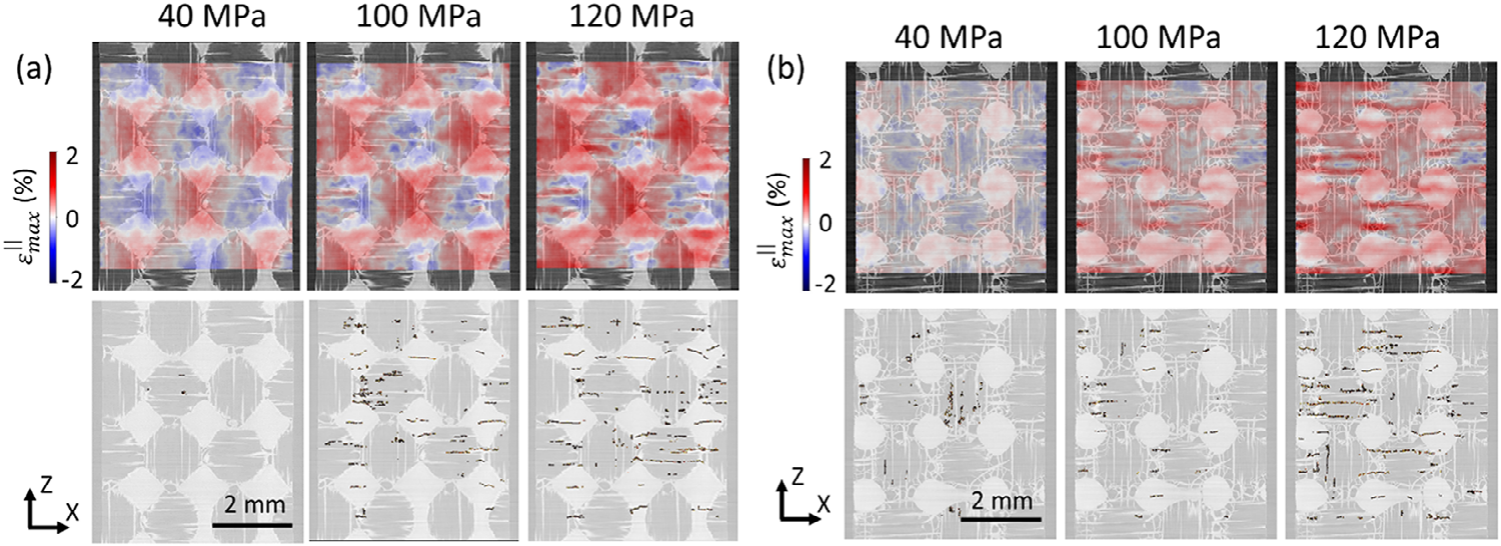
Maps of $\varepsilon_{max}^{\left|\right|}$ (top) and detected cracks (bottom) in the XZ‐plane superimposed onto the grayscale image for a) Batch‐A and b) Batch‐B. Videos are provided in Figures S2–S5 (Supporting Information) showing different cross sections of each sample.

**Figure 12:**
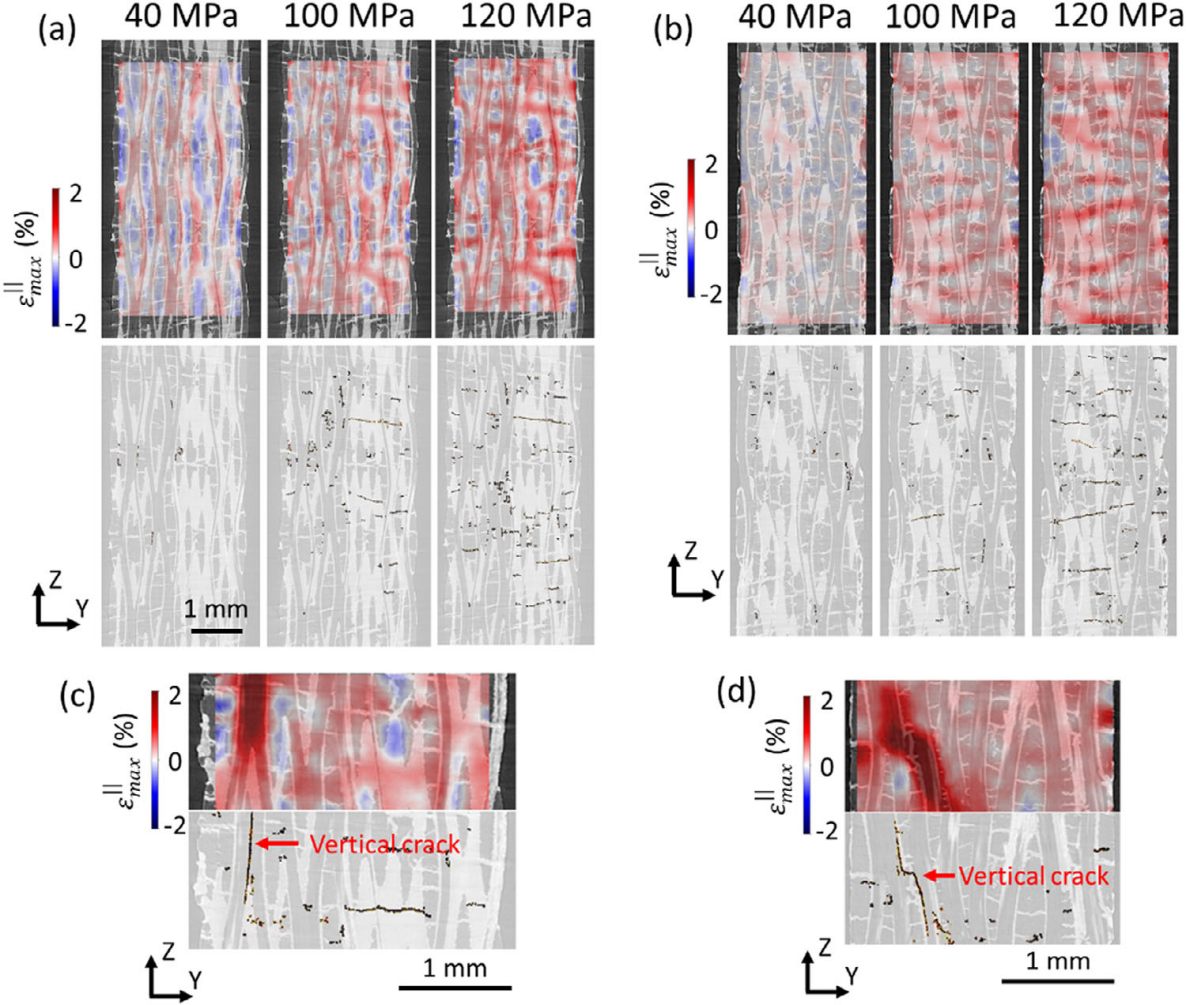
Maps of $\varepsilon_{max}^{\left|\right|}$ (top) and detected cracks (bottom) in the YZ‐plane superimposed onto the grayscale image for a) Batch‐A and b) Batch‐B. Example vertical cracks are shown in c) for Batch‐A and d) for Batch‐B at 120 MPa. Videos are provided in Figures S6–S9 (Supporting Information) showing different cross sections of each sample.

To visually correlate the strain and crack distributions with the C/C‐SiC microstructure, we superimpose the corresponding maps (dominant strains $\varepsilon_{max}^{\left|\right|}$ and detected cracks) over the grayscale reference image, as shown in **Figure** **11** for the XZ‐plane and in **Figure** **12** for the YZ‐plane.

Overall, the distributions of strain localization and cracks are strongly correlated with the composite microstructure. The strain distribution patterns in the two samples show a clear difference. As illustrated in Figure 11, Batch‐A exhibits a checkerboard‐type pattern corresponding well to the plain weave architecture, whereas Batch‐B displays a crack‐like pattern. Furthermore, Figure 12 reveals that in Batch‐B the regions of high‐magnitude positive strains remain aligned horizontally, while in Batch‐A these high‐strain zones “deviate” into the vertically oriented SiC‐Si skeletons. High‐magnitude negative strains are less frequent in Batch‐B compared to Batch‐A. The more complex strain distribution in Batch‐A implies a more effective 3D microstructural response to external load, leading to improved energy dissipation compared to Batch‐B.

Visually, most cracks primarily propagate in the horizontal/transverse direction (XY plane) along the SiC‐Si skeleton interfaces, and within the Si bulk regions. Both samples exhibit similar crack networks, although with subtle differences in morphology and density. It is also worth noting that vertical cracks are observed in both samples from the stress level of 100 MPa, suggesting local debonding between fiber bundles. While the vertical cracks remain straight lines in Batch‐A (Figure 12c), in Batch‐B, some vertical cracks appear to join with adjacent ones, forming a prominent stair‐like geometry (Figure 12d). These differences can be attributed to the microstructural difference: Batch‐B features a more tortuous microstructure with more severe fiber bundle waviness.

As the applied load increases, both samples show a progressive increase in crack density, with individual cracks becoming larger and more pronounced. Cracks appear to initiate at the SiC‐Si skeleton interfaces between fiber bundles, subsequently growing in the transverse direction (*x*‐axis) and through the thickness of the composite (*y*‐axis). Identifying precise damage initiation sites remains challenging due to the limited temporal resolution of in situ XCT. Future strategies could include faster scanning techniques, such as synchrotron‐based tomography, to improve temporal resolution. Alternatively, image‐based simulations using real microstructure geometries may help predict stress concentrations and potential initiation zones, offering a complementary numerical approach.^[^ ^37^ ^]^

### 3.3 Damage Evolution by AE Analysis

**Figure 13:**
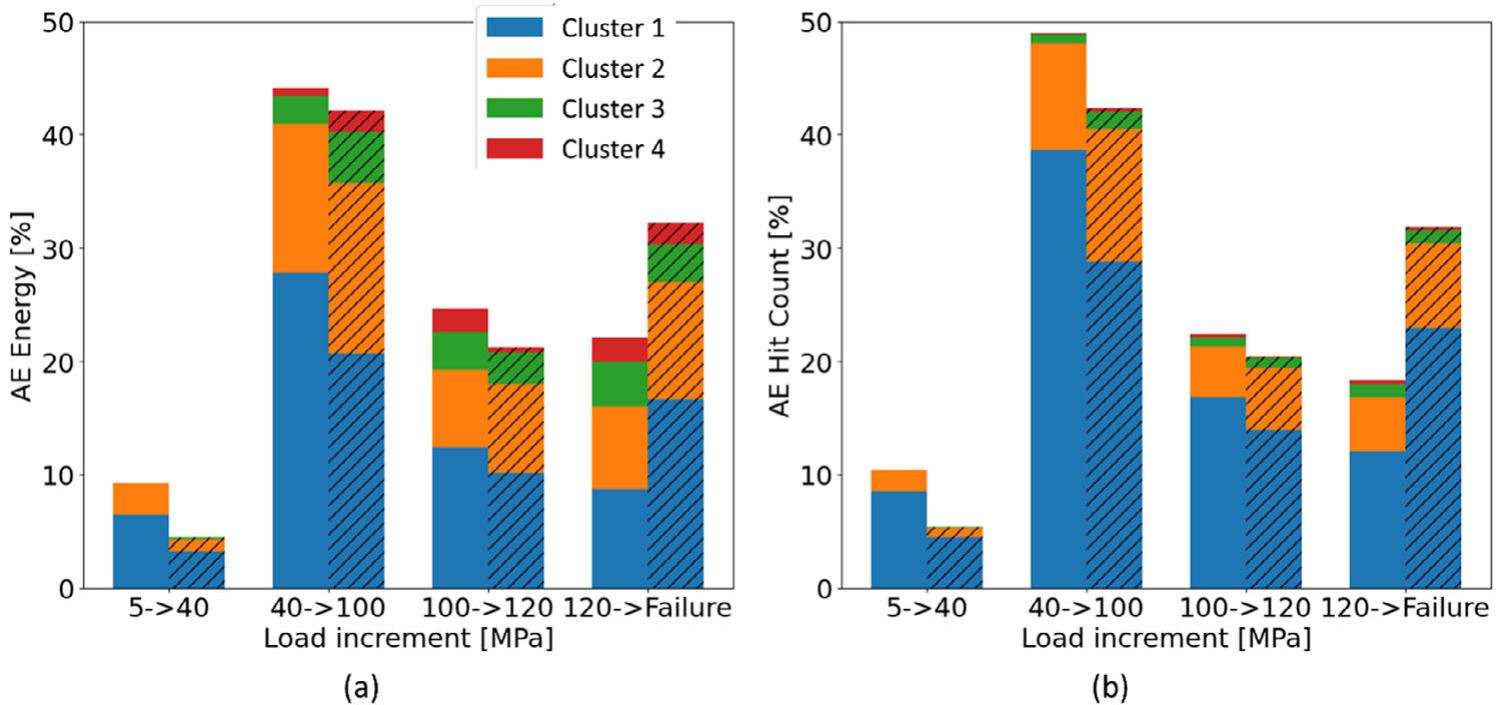
Percentages of acoustic emission energy a) and hit count b) emitted by different clusters at different load increments of the in situ tensile test. Each bar represents a load increment and is divided into colored segments corresponding to individual clusters. Hatching is used to distinguish Batch‐B.

The AE signal energy and hit count measured at different load increments are shown in **Figure** **13**. Normalization was performed by dividing the measured AE energy or hit counts of each sample by its respective total, accounting for sample‐to‐sample variations in AE signal numbers.

At the initial increment, up to 40 MPa, both samples exhibit limited AE activity, with most signals categorizing into Clusters 1 and 2. During this phase, Batch‐A demonstrates greater relative damage, particularly indicative of matrix cracking or the release of initial weak points. This loading range largely falls within the linear regime of the stress–strain curve, suggesting that these initial weak‐point fractures may not produce a measurable macroscopic change. The in situ XCT imaging reveals an opposite trend comparing the two samples at this stress level: more detectable cracks appear in Batch‐B than in Batch‐A. This discrepancy suggests that many of these low‐energy matrix and interface failures (Cluster 1&2) produce microcracks with small sizes and openings that are below the XCT resolution threshold. This is to be expected as most of them probably originate from internal residual stresses.

As the load increases from 40 to 100 MPa, AE signals from all clusters begin to emerge. This coincides with the onset of the macroscopic non‐linear regime (Figure 8). While Clusters 1 and 2 remain dominant in both samples, high‐frequency clusters (3 and 4) are slightly more pronounced in Batch‐B than in Batch‐A. This indicates that the Batch‐B microstructure incurred serious damage earlier, potentially contributing to its lower ultimate tensile strength. This earlier appearance of severe damage in Batch‐B is also underscored by the DVC strain distributions (Figures 11 and 12), showing that the crack‐like geometries appear in Batch‐B at 100 MPa, which is earlier than in Batch‐A.

During the 100‐>120 MPa increment, the trend reverses: Batch‐A emits a higher proportion of high‐frequency AE signals (Clusters 3 and 4) than Batch‐B, typically due to larger cracks formed in stiff constituents, as shown in Figures 11 and 12.

In the final increment leading to failure, Batch‐B generates significantly higher AE energy and hit counts associated with low‐energy signals (Clusters 1&2), while the levels of more energetic, high‐frequency signals (Clusters 3&4) remain comparable between the two samples. This final stage of damage could not be captured by in situ XCT, highlighting the value of AE monitoring as a complementary technique. The predominance of low‐energy signals in Batch‐B suggests that weak‐point fractures, such as matrix and interface damage, remain a major damage mode in the sample even up to ultimate failure.

To sum up the comparison based on Figures 11, 12, 13, early damage in Batch‐A dissipates more mechanical energy and makes more effective use of the reinforced microstructure at later load increments. In other words, the complex strain distribution triggered by the microstructure has a positive effect.

These observations highlight the inherent limitations of relying on either XCT or AE alone to characterize damage evolution in complex composite systems. XCT provides high spatial resolution for detecting and quantifying visible cracks, but it lacks temporal continuity and is insensitive to sub‐resolution damage, particularly during the early stages of loading. In contrast, AE offers real‐time monitoring of fracture events with high temporal resolution, capturing subtle damage processes as they occur, but it does not provide spatial information about the damage morphology. AE serves as a valuable complementary tool to in situ XCT, helping to validate and enhance the interpretation of damage mechanisms that may not be fully captured by imaging alone. It should, however, be noted that a one‐to‐one correlation between AE signals and XCT‐detected cracks cannot be established. This is primarily due to the possibility of multi‐phase crack propagation, i.e., one AE signal may originate from a crack propagating multiple phases (from interface to matrix, for instance). Additionally, the temporal resolution of XCT is much lower than AE monitoring since scans are only performed at specific load levels. To address this complexity, further refinement of the AE signal clustering technique may be beneficial.^[^ ^33^ ^]^

### 3.4 Summary of Damage Mechanisms and Comparison of the Two Samples

**Figure 14:**
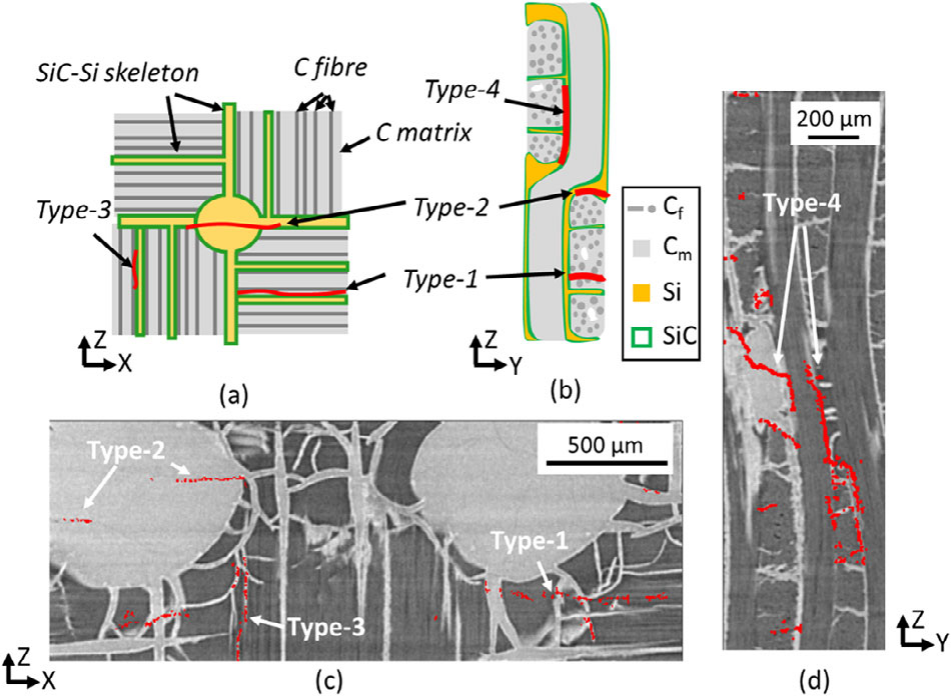
a,b) Illustration of the detected damage mechanisms in the C/C‐SiC composites. c,d) Detected cracks superimposed onto the reference grayscale image of Batch‐B, showing different crack types.

**Figure** **14** illustrates the damage mechanisms observed in the C/C‐SiC composites. We summarize the detected cracks into four different types according to their locations and orientations.

1. Type‐1 cracks grow in the transverse direction (perpendicular to the tensile load). They appear either around the SiC‐Si skeleton or inside the C matrix.
2. Type‐2 cracks also grow in the transverse direction, but they are inside the Si bulks. Visual inspection suggests that these cracks are mainly related to further opening and propagation of the pre‐existing cracks in the as‐received materials (Figure 14.c).
3. Type‐3 cracks grow in the vertical direction (parallel to the load) and open in the transverse direction. These cracks mostly appear around the SiC‐Si skeleton.
4. Type‐4 cracks also grow in the vertical direction, but open in the through‐thickness direction. They are mainly located between 0° and 90° fiber bundles. They can grow into larger geometries, especially in Batch‐B forming stair‐like delaminations, as shown in Figure 14d.

Based on the XCT observation (Figures 10, 11, 12) and the AE monitoring (Figure 13), we postulate that the early loading stage primarily activates the small type‐1 and type‐3 cracks, emitting low‐frequency, low‐energy AE signals. These cracks are likely initiated at pre‐existing weak points induced during fabrication. As the load increases beyond the linear regime, additional damage mechanisms begin to emerge, with larger (XCT detectable) type‐2 cracks becoming particularly prominent, emitting “louder” AE signals. Connecting to pre‐existing cracks, the detection of type‐2 cracks indicates that Si bulks started to carry more and more stress as the load increased. In the late load stage up to failure, damage is featured by the significant growth and coalescence (especially in Batch‐B) of type‐4 cracks. These cracks reflect progressive local debonding between fiber bundles, which eventually evolve into larger delaminations. The type‐4 cracks may not directly correspond to the high‐frequency, high‐energy AE signals (Cluster 4), but can be a consequence of the latter, indicating severe damage, such as the fracture of SiC and fibers.

A comparison between the two samples further illustrates the impact of microstructural features on damage evolution. Batch‐B, which was processed with higher compaction pressure, exhibited more isolated Si bulk regions and more tortuous fiber bundle paths. This microstructural configuration was found to be less effective in resisting damage accumulation. Specifically, Batch‐B showed more load‐driven strain localization and weaker microstructural modulation of deformation, as evident from Figures 11 and 12. Additionally, more high‐frequency and high‐energy AE signals (Clusters 3 and 4 in Figure 14) appeared at the intermediate stress level (40‐>100 MPa) in Batch‐B than in Batch‐A, which suggests early critical damage in Batch‐B, possibly due to more severe waviness of the fiber bundles.

### 3.5 Remark on Statistical Representativeness

While conducting additional in situ tests on more samples would have been ideal, this is often limited by the available resources and time frame. Nonetheless, the in situ experimental results showed strong agreement with macroscopic tests, which were repeated five times and consistently demonstrated the stress–strain behavior across both batches. This repeatability serves as indirect validation of the composite behavior and reinforces the reliability of the in situ findings. Furthermore, each in situ test provides full‐field measurements of microstructural changes, offering rich, statistically meaningful insights into the correlation between microstructure and damage evolution. In effect, the structure‐damage relationship is observed across multiple sites within a single sample, encompassing repeated patterns of the woven fabric. This yields far more comprehensive information than localized techniques such as nano‐indentation, which require numerous repetitive tests to achieve similar coverage.

## 4 Conclusion

By integrating in situ X‐ray computed tomography (XCT) with the acoustic emission (AE) technique, this study provides the first comprehensive characterization of how residual silicon interconnectivity influences damage evolution in C/C‐SiC composites fabricated using a thermoplastic precursor. The quantitative analysis of in situ XCT data, combined with AE signal clustering, offered novel insights into the different damage mechanisms. Microcracks were categorized into four distinct types, enabling a more nuanced understanding of failure processes that macroscopic methods could not capture.

A comparative analysis of two composite systems highlights the crucial role of microstructure in governing damage progression. The findings reveal that enhanced interconnectivity of the silicon bulk region promotes more effective utilization of fiber reinforcement. This is evidenced by the strain distribution that is more strongly influenced by the microstructure and a more gradual development of microcracks in the Batch‐A composite (which has more interconnected silicon). This work exemplifies the unique capability and potential of quantitative XCT, particularly when combined with AE techniques, for advanced damage analysis in CMCs. The above conclusions about the role of silicon bulk interconnectivity would not be accessible through conventional testing approaches.

This methodology has potential for integration with image‐based modeling^[^ ^38^ ^]^ to determine stress distributions within the real microstructure at various loading stages. The experimentally detected cracks can be used either to validate numerical predictions (if a robust damage model is implemented) or as input geometries to investigate their influence on stress redistribution under the most realistic situation.

## Conflict of Interest

The authors declare no conflict of interest.

## Supporting information

Supporting Information

Supporting Video 1

Supporting Video 2

Supporting Video 3

Supporting Video 4

Supporting Video 5

Supporting Video 6

Supporting Video 7

Supporting Video 8

## Figures and Tables

**Figure A1:**
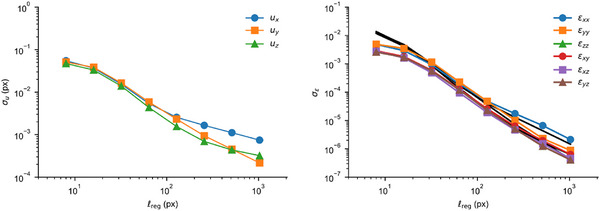
Estimated uncertainty for Batch A as a function of regularization length (mesh size fixed to 16 voxels): Left – displacement uncertainty; Right–Strain uncertainty (black lines correspond to the approximation $\sigma_{\varepsilon }=\frac{2\sigma_{u}}{\ell_{reg}}$).

**Figure A2:**
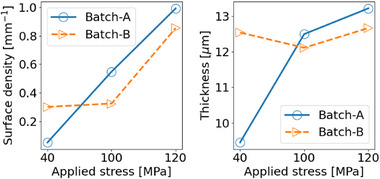
Crack surface density (left) and average crack thickness (right) measured at different stress levels in the two samples. The crack surface density was calculated as the ratio between the measured crack surface area (mm^2^) and the total volume of each sample (mm^3^).

## Data Availability

The data that support the findings of this study are openly available in Zenodo at [https://doi.org/10.5281/zenodo.16887072], reference number [0].

## Appendix A DVC Uncertainty Analysis

### A.1

As shown in Ref. [39] displacement uncertainty in global DIC/DVC approaches can be estimated through its covariance matrix as $\Sigma_{u}=2\sigma_{I}^{2}M_{DIC}^{-1}$ where $\sigma_{I}$ is the intrinsic (Gaussian) noise of the images and $M_{DIC}=\int_{\Omega }\left(\nabla I\cdot \phi_{i}\right)\left(\nabla I\cdot \phi_{j}\right)$ is the Hessian matrix resulting from the image gradient ∇*I* and the FE shape functions ϕ_*i*_. Thus, the effective DVC uncertainty depends on both the choices of discretization and image noise. Regularization can help reduce the uncertainty,^[^ ^40^ ^]^ as implemented in this work.

The FE mesh size should not be too small with respect to the texture of the image to capture “enough” gradient (even if it could be mitigated using regularization). This minimal size is also a practical limit on the computational effort required to solve the DVC problem. As a result, we fixed the mesh size at 16 voxels.

Ideally, DVC uncertainty should be estimated using two or more scans of the sample in a fixed loading state, applying a rigid motion between the scans. This captures both sources of uncertainty, but it requires a greater experimental effort. When only one scan of the reference state is available, a virtual image can be created by artificially shifting the reference image and adding Gaussian noise to simulate acquisition conditions.^[^ ^27^ ^]^

In our study, a virtual image was obtained by shifting the reference volume by 0.5 voxels in each direction.^[^ ^41^ ^]^ The intrinsic noise of the reference volume was estimated to be ≈8 gray levels, i.e., ≈3% of the 8‐bit dynamic range (0–255). Consequently, an additional zero‐mean Gaussian noise with a standard deviation of 8 was added to the moved volume. The DVC registration was then performed with descreasing regularization length from 1024 to 8 voxels.

The displacement uncertainty was quantified as the standard deviation σ_*u*_ of the computed displacement field (shifted by ‐0.5).^[^ ^27^ ^]^ Results for Batch‐A (Figure A1‐left) show that uncertainty decreases with increasing regularization length, confirming the theory.^[^ ^27^ ^]^ It is worth noting that DVC indeed allows for measuring sub‐voxel displacement, even in the absence of dedicated speckle, as in DIC.

Uncertainty in strain measurement can be either derived from displacement using the FE mesh, or calculated from the displacement uncertainty as $\sigma_{\varepsilon }=\frac{2\sigma_{u}}{\ell_{reg}}$. The results are plotted in Figure A1‐right. Once again, we obtain the expected power‐law decrease of the strain uncertainty with increasing regularization length. Note that the gauge length to be considered for the strain uncertainty is not the mesh size per se, but rather the regularization length, as it will impose the spatial variation of the displacement.

The choice of the regularization length is a compromise between the spatial resolution of the displacement (strain) field and the corresponding uncertainty. For example, to enhance the detection of strain localization and/or cracks, it is necessary to use a moderate regularization length while accepting higher uncertainty in the remainder of the volume. Accordingly, we selected 32 voxels as the regularization length.

## Appendix B Crack Quantification

### B.1

Using the crack quantification method from Ref. [32], we obtain the evolution of crack surface density and average crack thickness as functions of applied stress (Figure A2). In the early loading stage, Batch‐B exhibits significantly higher values for both crack surface density and thickness, suggesting more early‐activated weak points, which is likely due to its higher compaction pressure during fabrication. However, the measurements should be interpreted cautiously, as image resolution, noise, and artefacts may have obscured smaller cracks. This limitation may explain the slight drop in average crack thickness in Batch‐B at 100 MPa: at 40 MPa, only thicker cracks were visible, but as the load increased, thinner cracks began to open and became detectable, lowering the average thickness.
