# Supplementary material for: In Situ X‐Ray Tomography and Acoustic Emission Monitoring of Damage Evolution in C/C‐SiC Composites Fabricated by Liquid Silicon Infiltration
Source: Adv Sci (Weinh). 2025 Nov 13:e16200. Online ahead of print. doi: 10.1002/advs.202516200 (PMC13325554; doi:10.1002/advs.202516200)
Supplement: Supplementary file 1 — Supporting Information [file ADVS-9999-e16200-s001.docx]

**S1**. Histograms of the maximum principal strains $\varepsilon_{max}$ in the two C/C-SiC of the present study at different load increments (a), compared to the results from a previous paper on a different C/C-SiC material [1].


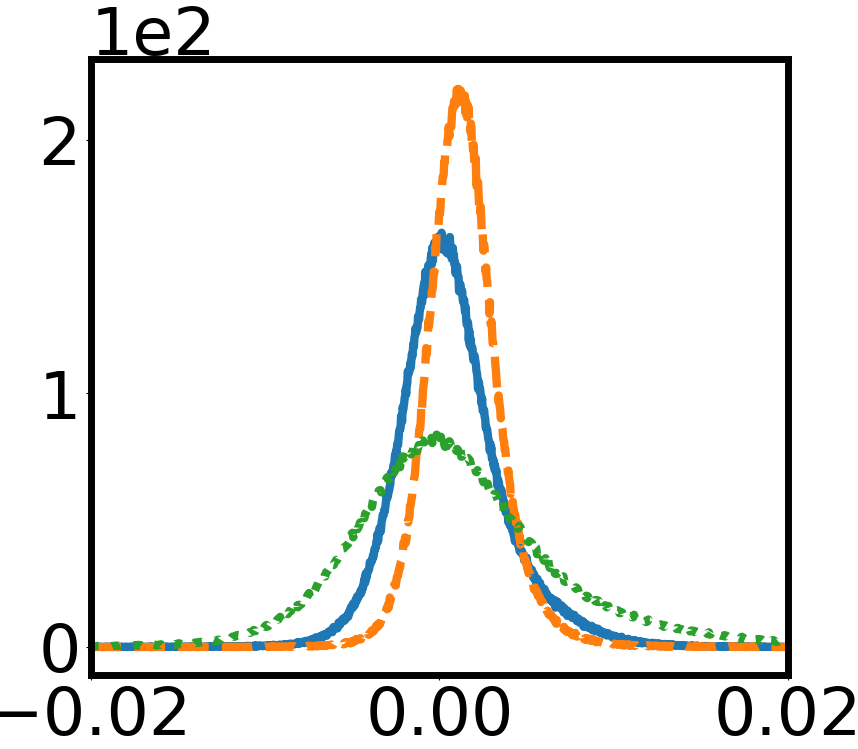

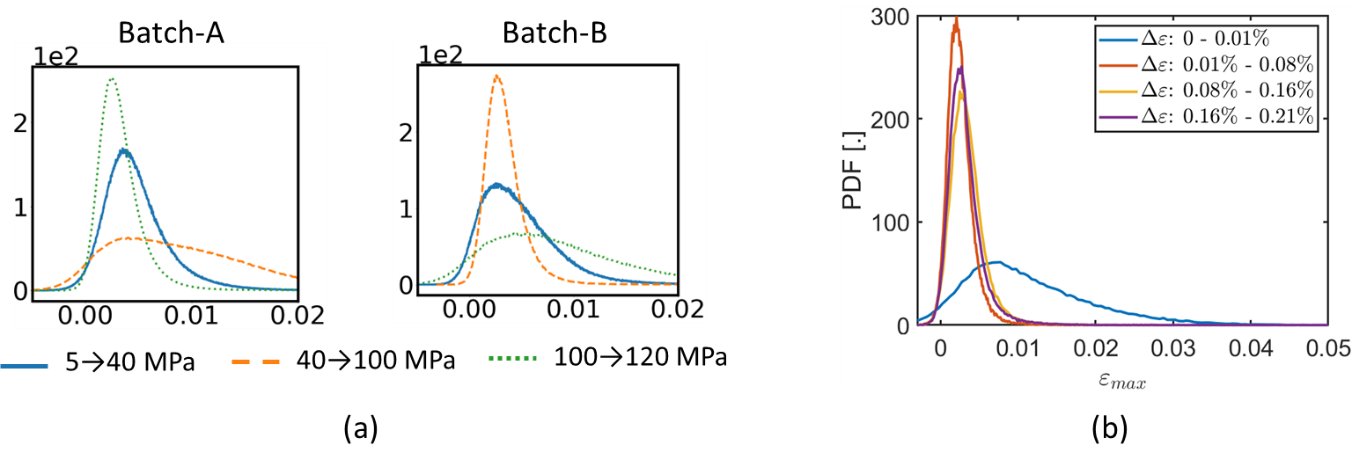


The main difference between the present material (made in Bayreuth) and the previous one (made in DLR) is the use of precursor in the CFRP before pyrolysis: the Bayreuth material used thermoplastic matrix hence leading to Si bulks in the final composites, whereas the DLR material used phenolic resin (thermoset) matrix hence minimising the residual silicon in the final product. A clear difference can be seen from this comparison, in terms microstructural response to tensile load – very large strain values were observed at the first loading step in the DLR materials, which was believed to be related to initial weak points from the LSI process. These weak points seemed to be depleted due to the fact that strains were mostly reduced at later load increments. In contrast, the Bayreuth material exhibited a more gradual change of the strain distribution. Although the first load increment created larger tails in the histograms (indicating greater strains), the mode remain relatively low. Therefore, the weak-point depletion does not hold for the Bayreuth material, meaning that the “weak points” survive from initial low-level loads and are gradually fractured until the ultimate failure.

1. Chen, Y., et al., *In situ X-ray tomography characterisation of 3D deformation of C/C-SiC composites loaded under tension.* Composites Part A: Applied Science and Manufacturing, 2021. **145**: p. 106390.
