# Supplementary figures and images for: In Situ X‐Ray Tomography and Acoustic Emission Monitoring of Damage Evolution in C/C‐SiC Composites Fabricated by Liquid Silicon Infiltration
Source: Adv Sci (Weinh). 2025 Nov 13:e16200. Online ahead of print. doi: 10.1002/advs.202516200 (PMC13325554; doi:10.1002/advs.202516200)

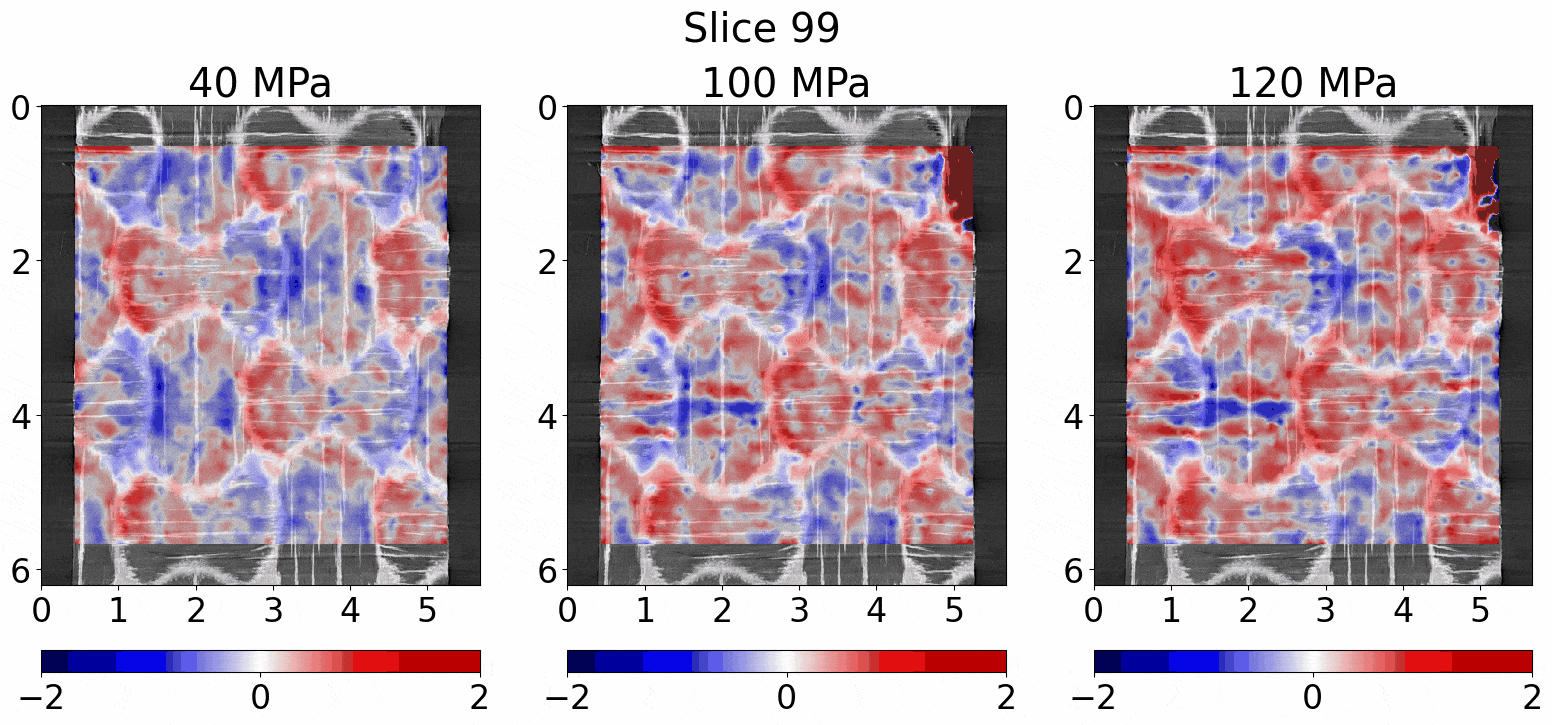

Supplement: Supplementary file 2 — Supporting Video 1 [file ADVS-9999-e16200-s003.gif]

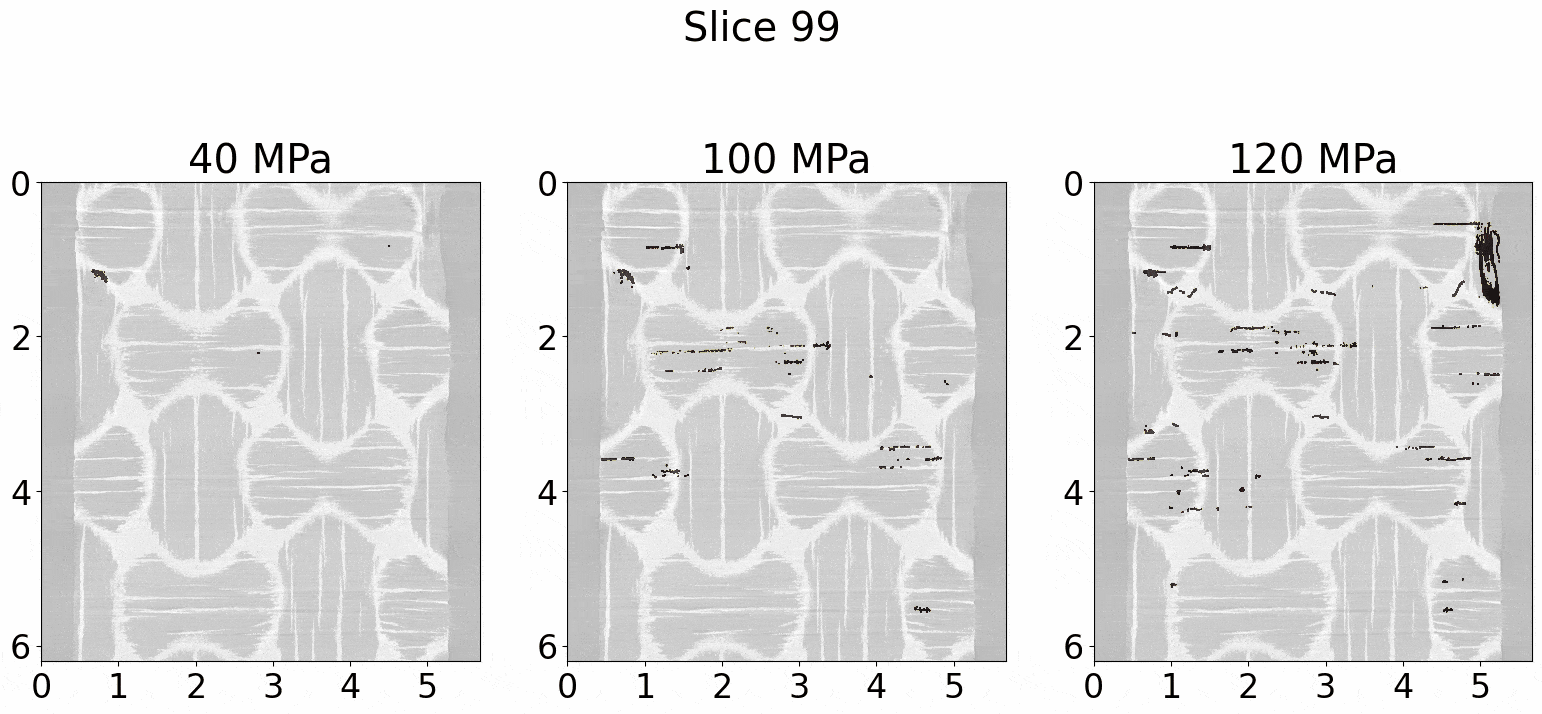

Supplement: Supplementary file 3 — Supporting Video 2 [file ADVS-9999-e16200-s004.gif]

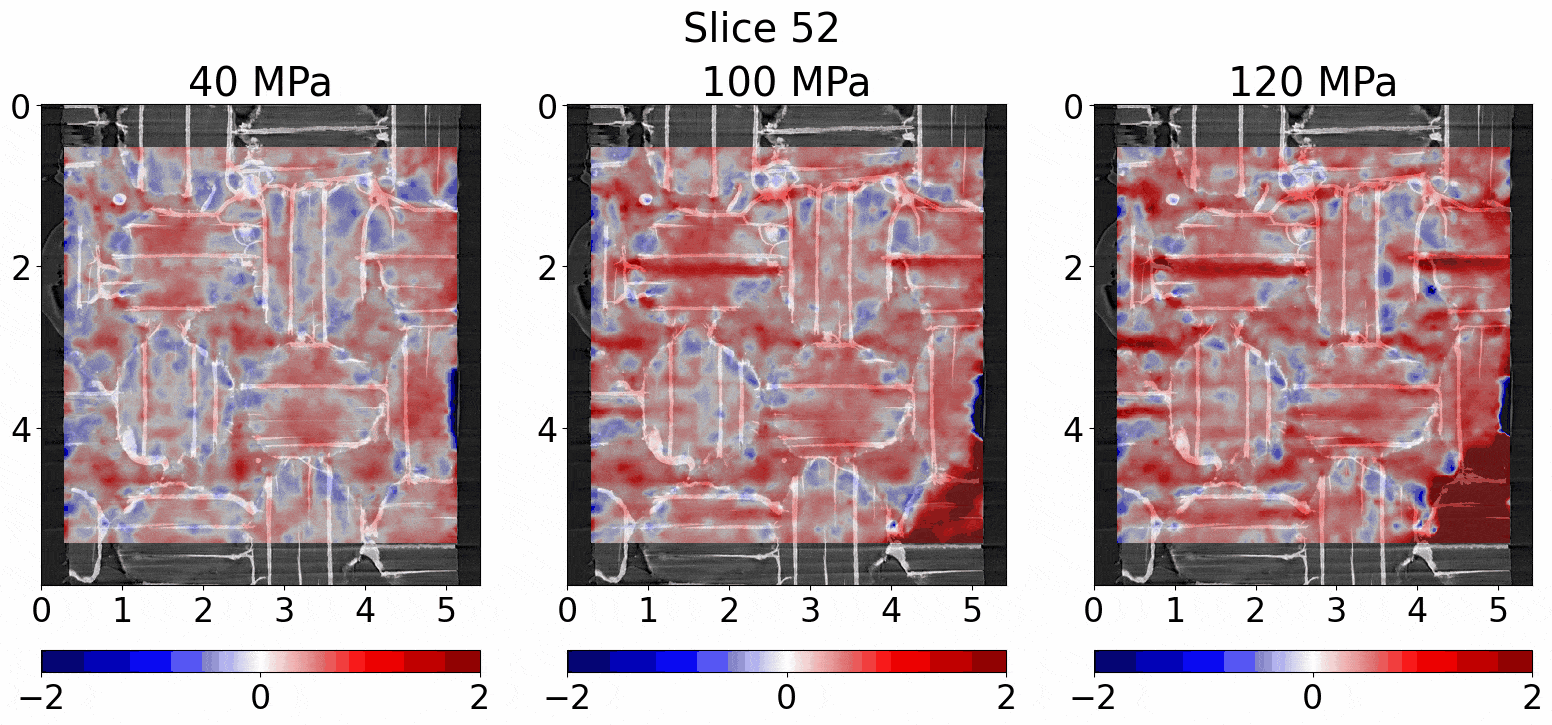

Supplement: Supplementary file 4 — Supporting Video 3 [file ADVS-9999-e16200-s008.gif]

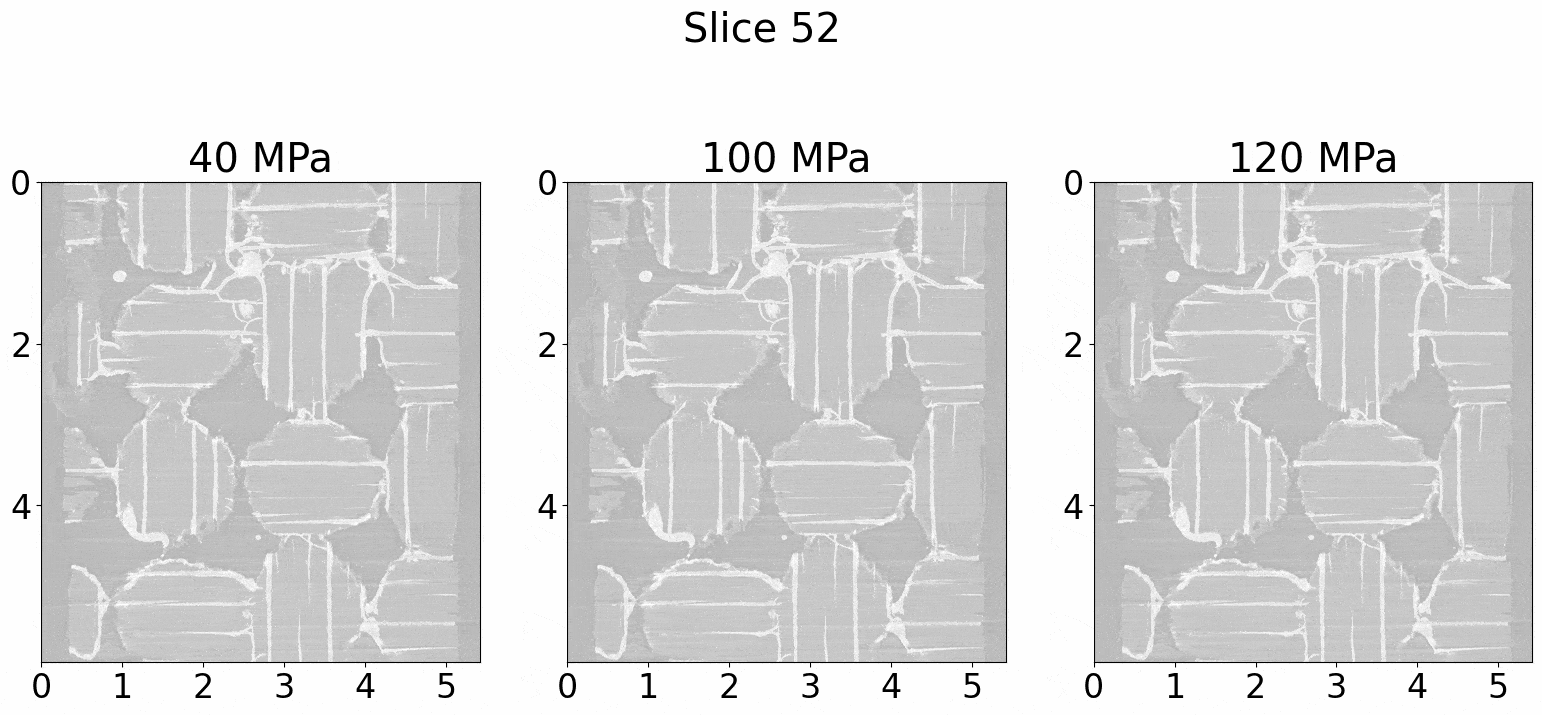

Supplement: Supplementary file 5 — Supporting Video 4 [file ADVS-9999-e16200-s006.gif]

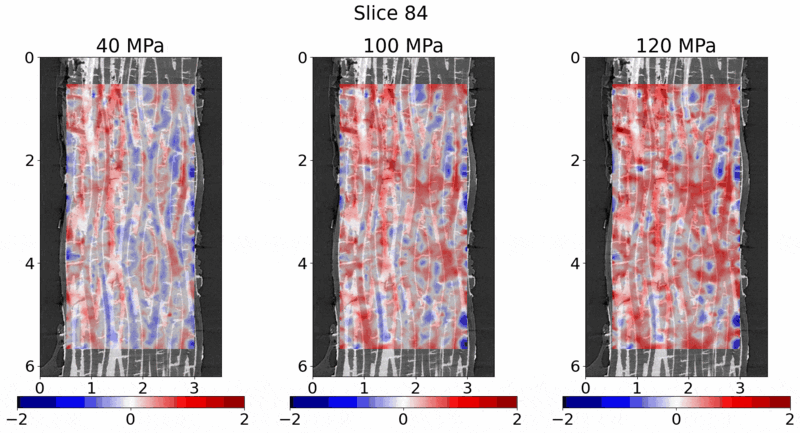

Supplement: Supplementary file 6 — Supporting Video 5 [file ADVS-9999-e16200-s009.gif]

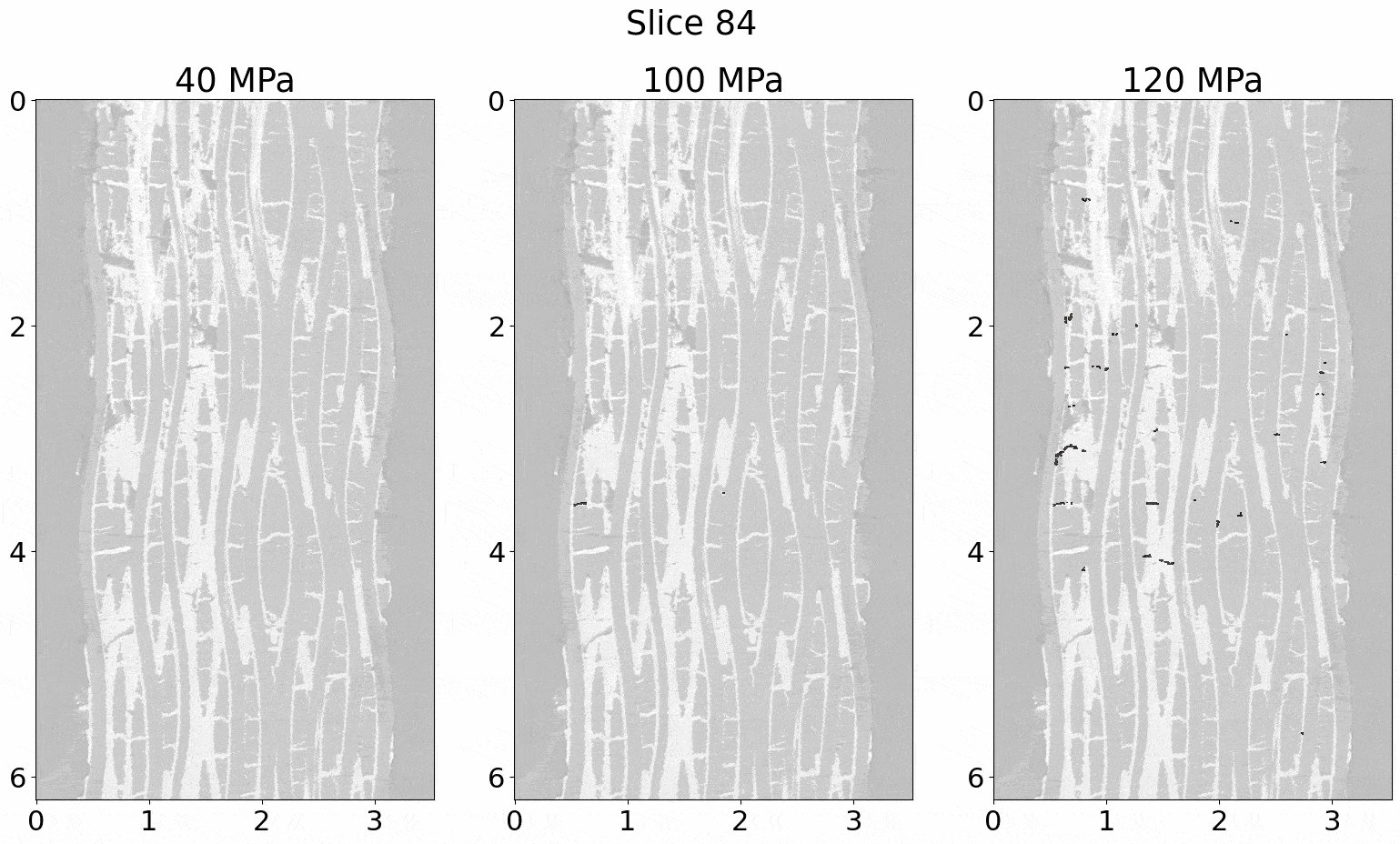

Supplement: Supplementary file 7 — Supporting Video 6 [file ADVS-9999-e16200-s005.gif]

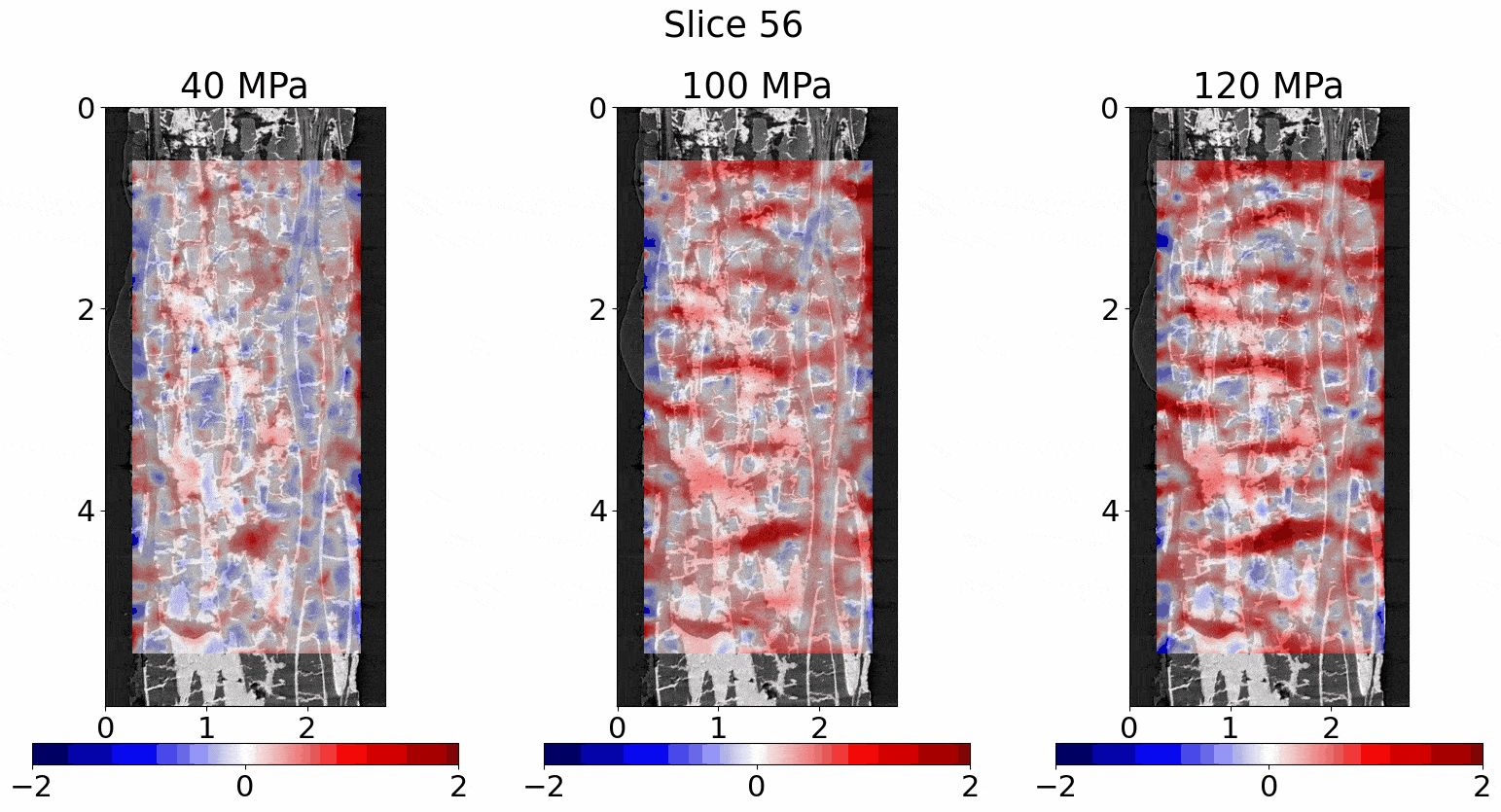

Supplement: Supplementary file 8 — Supporting Video 7 [file ADVS-9999-e16200-s007.gif]

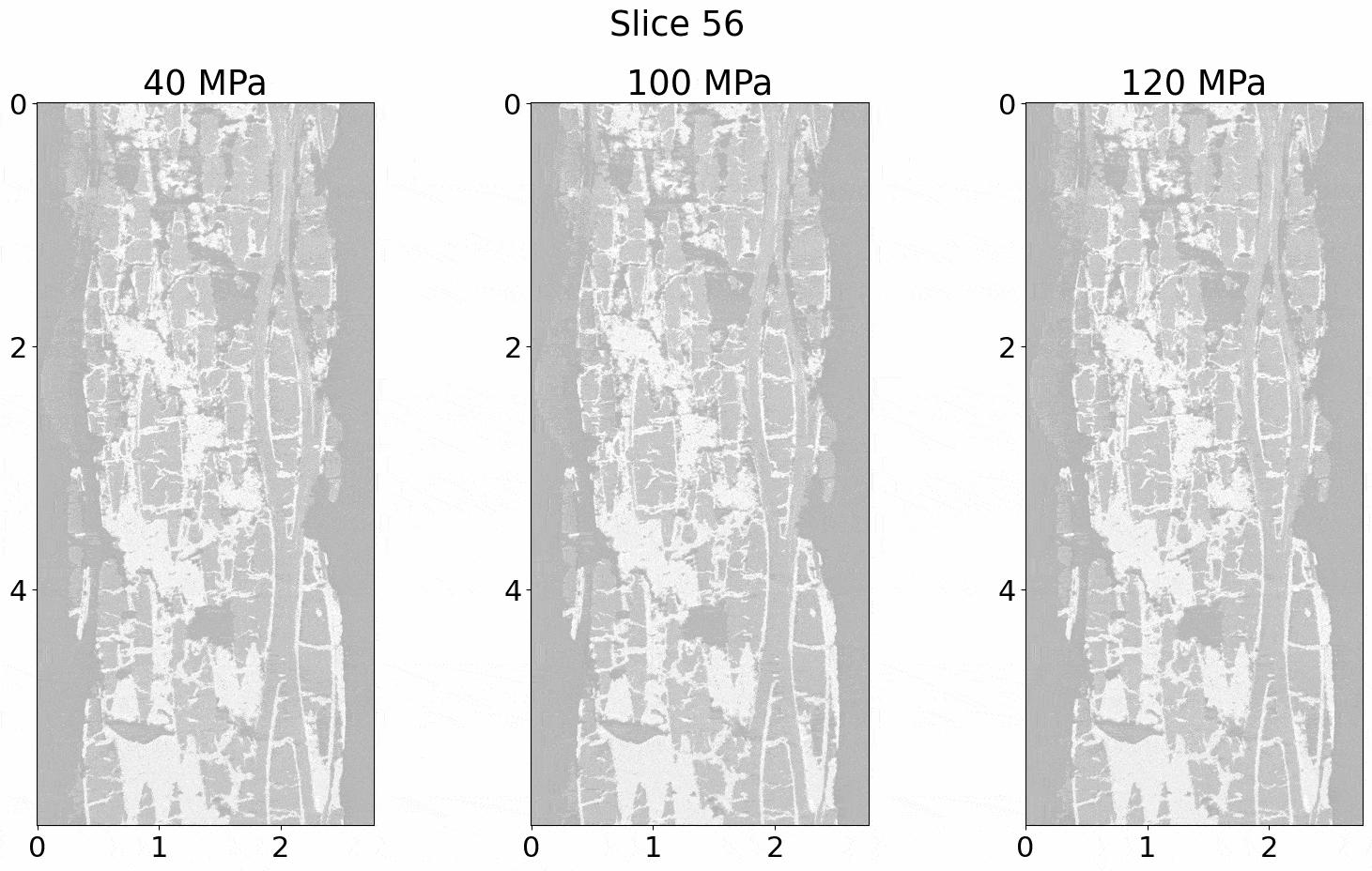

Supplement: Supplementary file 9 — Supporting Video 8 [file ADVS-9999-e16200-s002.gif]
